# Key Role of Splenic Myeloid DCs in the IFN-αβ Response to Adenoviruses *In Vivo*

**DOI:** 10.1371/journal.ppat.1000208

**Published:** 2008-11-14

**Authors:** György Fejer, Lisa Drechsel, Jan Liese, Ulrike Schleicher, Zsolt Ruzsics, Nicola Imelli, Urs F. Greber, Simone Keck, Bernd Hildenbrand, Anne Krug, Christian Bogdan, Marina A. Freudenberg

**Affiliations:** 1 Max-Planck-Institute for Immunobiology, Freiburg, Germany; 2 Institute of Medical Microbiology and Hygiene, University Clinic of Freiburg, Freiburg, Germany; 3 Microbiology Institute - Clinical Microbiology, Immunology and Hygiene, University Clinic of Erlangen, Erlangen, Germany; 4 Max von Pettenkofer Institut für Virologie, Ludwig-Maximilians-Universität München, Munich, Germany; 5 Institute of Zoology, University of Zurich, Zürich, Switzerland; 6 Tumor Biology Center, Freiburg, Germany; 7 Department of Internal Medicine, Technical University of Munich, Munich, Germany; Oregon Health & Science University, United States of America

## Abstract

The early systemic production of interferon (IFN)-αβ is an essential component of the antiviral host defense mechanisms, but is also thought to contribute to the toxic side effects accompanying gene therapy with adenoviral vectors. Here we investigated the IFN-αβ response to human adenoviruses (Ads) in mice. By comparing the responses of normal, myeloid (m)DC- and plasmacytoid (p)DC-depleted mice and by measuring IFN-αβ mRNA expression in different organs and cells types, we show that *in vivo*, Ads elicit strong and rapid IFN-αβ production, almost exclusively in splenic mDCs. Using knockout mice, various strains of Ads (wild type, mutant and UV-inactivated) and MAP kinase inhibitors, we demonstrate that the Ad-induced IFN-αβ response does not require Toll-like receptors (TLR), known cytosolic sensors of RNA (RIG-I/MDA-5) and DNA (DAI) recognition and interferon regulatory factor (IRF)-3, but is dependent on viral endosomal escape, signaling via the MAP kinase SAPK/JNK and IRF-7. Furthermore, we show that Ads induce IFN-αβ and IL-6 *in vivo* by distinct pathways and confirm that IFN-αβ positively regulates the IL-6 response. Finally, by measuring TNF-α responses to LPS in Ad-infected wild type and IFN-αβR^−/−^ mice, we show that IFN-αβ is the key mediator of Ad-induced hypersensitivity to LPS. These findings indicate that, like endosomal TLR signaling in pDCs, TLR-independent virus recognition in splenic mDCs can also produce a robust early IFN-αβ response, which is responsible for the bulk of IFN-αβ production induced by adenovirus *in vivo*. The signaling requirements are different from known TLR-dependent or cytosolic IFN-αβ induction mechanisms and suggest a novel cytosolic viral induction pathway. The hypersensitivity to components of the microbial flora and invading pathogens may in part explain the toxic side effects of adenoviral gene therapy and contribute to the pathogenesis of adenoviral disease.

## Author Summary

Adenoviruses (Ads) are important pathogens and promising vectors for gene therapy applications. In the course of adenoviral infections innate immune responses are activated, which can be beneficial for the antiviral host defense but also detrimental if activated in a deregulated manner. Type I IFNs are crucial for the innate immune control of various viral infections in the mammalian host. So far, the early, systemic release of IFN-αβ during viral infections has been attributed to specialized immune cells, the plasmacytoid dendritic cells. Here, in a mouse infection model, we show that wild type Ads, as well as adenoviral vectors, elicit rapid IFN-αβ production almost exclusively in another cell population, the splenic myeloid dendritic cells. This IFN-αβ storm depends on viral escape from endosomes to the cytosol and the requirements of the response are suggestive of a novel viral induction pathway. Furthermore, we show that virus induced IFN-αβ is the key mediator of Ad-induced hypersensitivity to the cytokine-inducing and toxic activity of lipopolysaccharide, a common constituent of Gram-negative bacteria. Since these bacteria comprise several commensals and pathogens, enhanced susceptibility to lipopolysaccharide may contribute to toxic reactions observed during adenoviral gene therapy and to the clinical symptoms of adenoviral diseases.

## Introduction

Adenoviruses (Ads) cause mild disease in humans, but are hazardous pathogens in immuno-compromised individuals [1]. Human Ads are dsDNA viruses grouped into six species. Species A, C, D, E, and F and species B Ads use different infectious entry pathways [2]. Human Ads enter mouse cells and express their early genes; however, the virus genome is not replicated and no viral progeny is made during the infection of mouse cells *in vitro* or *in vivo* [3],[4]. Furthermore, early viral gene expression can be abolished by UV-inactivation and well-defined mutants with defects of viral early genes or viral entry are available [3],[5]. Thus, the effects elicited by different components of the virus-host interaction preceding viral replication can be accurately evaluated.

Ads transduce many different cell types and can be produced *in vitro* in sufficiently high amounts for *in vivo* administration. While these properties make them attractive for gene therapy applications, they can also trigger a severe systemic toxic reaction [6],[7]. Upregulation of inflammatory mediators, including cytokines and chemokines such as IL-1, IL-6, IL-8, IL-12, macrophage inhibitory protein-1/2, tumor necrosis factor-α (TNF) and recently also type I IFN has been observed in experimental and clinical infections with wt as well as with recombinant Ads [6],[7],[8],[9],[10].

Type I IFNs represent one of the host's most important antiviral defense mechanisms. The type I IFN family comprises different IFN-α subtypes, a single IFN-β and other less well characterized proteins [11]. All IFN-α species and IFN-β interact with the same IFN-αβ cellular receptor, the activation of which mediates a wide range of direct and indirect innate antiviral or antimicrobial effects and modulates the antiviral adaptive immune response [12],[13],[14].

At present, two main mechanisms of type I IFN induction by viruses resulting from the extracytoplasmic or cytoplasmic virus recognition, respectively, are known [12],[13],[14],[15]. The extracytoplasmic induction is initiated by triggering the surface-expressed transmembrane protein toll-like receptor (TLR) 4 with certain non-nucleic viral constituents [16],[17],[18] or upon recognition of viral nucleic acids in the endosomes of specialized cells (dendritic cells and macrophages) *via* different members of the TLR family. These include TLR3, TLR7/TLR8 and TLR9, sensing dsRNA, ssRNA and CpG DNA, respectively. For IFN-αβ induction TLR3 and TLR4 signal through the adaptor molecule TIR domain-containing adaptor protein inducing interferon β (TRIF). This results in the activation of interferon regulatory factor (IRF)-3. TLR7, 8 and 9 signal through the adaptor molecule Myeloid differentiation factor 88 (MyD88). An important result of the MyD88-mediated pathway is the activation of IRF-7 (but not of IRF-3), which together with the transcription factors NF-kB and AP-1 initiates the induction of both the IFN-α and IFN-β genes [13],[15]. This induction pathway is responsible for the strong, early IFN-αβ response to several replicating and inactivated viruses in pDCs, which express preferentially TLR7 and TLR9 [19],[20],[21].

The “classical” cytosolic pathway is the major IFN-αβ producing mechanism in cells other than pDCs [22],[23]. Signal transduction leading to type I IFN gene induction is initiated by the recognition of intracellular virus-associated molecular patterns. dsRNA and 5′-triphosphate RNA produced during viral replication are sensed by the RNA helicases RIG-I and MDA-5 [23],[24],[25],[26],[27],[28]. This pathway has been extensively studied, mainly in virus-infected fibroblasts. Triggering of the aforementioned RNA helicases leads to the activation of the transcription factors NF-kB, IRF-3 and IRF-7 that are important for the induction of IFN-αβ and proinflammatory cytokines, including IL-6. In the cytosolic pathway, type I IFN gene induction is a sequential event and both IRF-3 and IRF-7 were shown to be important in the early phase when mostly IFN-β is produced. The late phase of the IFN-αβ response is regulated by positive feedback via the increased levels of IRF-7 elicited by IFN-β production during the early phase [29],[30],[31]. In addition to fibroblasts, the potential of mDCs and macrophages to produce significant amounts of type I IFNs in response to viral replication has been demonstrated *in vitro* [32],[33]; however, *in vivo* the specific contribution of these cells to systemic levels of IFN-αβ is not well documented.

Recently, detection of bacterial DNA in cells infected with *L. monocytogenes* and recognition of transfected B-DNA has been shown to trigger IFN-β production. This type of response strictly requires IRF-3 [15],[34],[35],[36],[37],[38]. Such a sensing system has been suggested to represent a further mechanism of cytosolic DNA virus recognition [14],[15] and the Z-DNA binding protein 1 (Zbp1, also referred as DNA-dependent activator of IFN regulatory factors, DAI) was shown to be a candidate DNA sensor in this pathway [39]. Notably however, in a follow-up study the same group found a critical role for DAI in L-929 cells but not in mouse embryonic fibroblasts (MEFs) [40]. Furthermore, recent experiments with Zbp1/DAI knockout mice did not show the essential role of Zbp1/DAI in the induction of innate and adaptive responses to B-DNA *in vivo* and in macrophages, dendritic cells and MEFs *in vitro* [41].

The induction of type I IFN in Ad-infected mice has been recently studied [10],[42] and associated with both the extracytoplasmic and intracytoplasmic pathways. It was claimed that a part of the IFN-αβ response is initiated by TLR9 and MyD88 signaling in pDCs and another part by cytosolic DNA recognition in non-pDCs. However, the identification of IFN-αβ producing cell types directly in infected mice was not carried out. In the present study we investigated the IFN-αβ responses of Ad-infected mice and showed that the bulk of the *in vivo* induced IFN-αβ is produced by splenic mDCs. Furthermore, we found that TLRs, including TLR9 play no major role. The Ad-elicited IFN-αβ response required viral endosomal escape, suggesting a cytosolic induction pathway. Surprisingly however, the induction was independent of IRF-3 and dependent on stress-activated protein kinase/c-Jun NH2-terminal kinase (SAPK/JNK) activity, which is in contrast to the known induction mechanism initiated through cytosolic DNA recognition. Instead, the induction required IRF-7, and a positive feedback regulation *via* the type I IFN receptor. Although this does not exclude a role for cytosolic nucleic acid sensors, our data do not support the involvement of MDA-5, RIG-I and Zbp1/DAI in the induction of the IFN-αβ response to Ad. Furthermore, our results reveal distinct mechanisms in the induction of IFN-αβ and IL-6 by Ad. Finally, we show that Ad-induced IFN-αβ is a key mediator of hypersensitivity to bacterial lipopolysaccharides in infected mice. Enhanced susceptibility to LPS and to other microbial inducers of inflammation may contribute to toxic reactions observed during adenoviral gene therapy and to the clinical symptoms of adenoviral diseases.

## Results

### Induction of IFN-αβ by adenovirus in mice is elicited by viral entry and is inhibited by viral early gene expression

**Figure 1:**
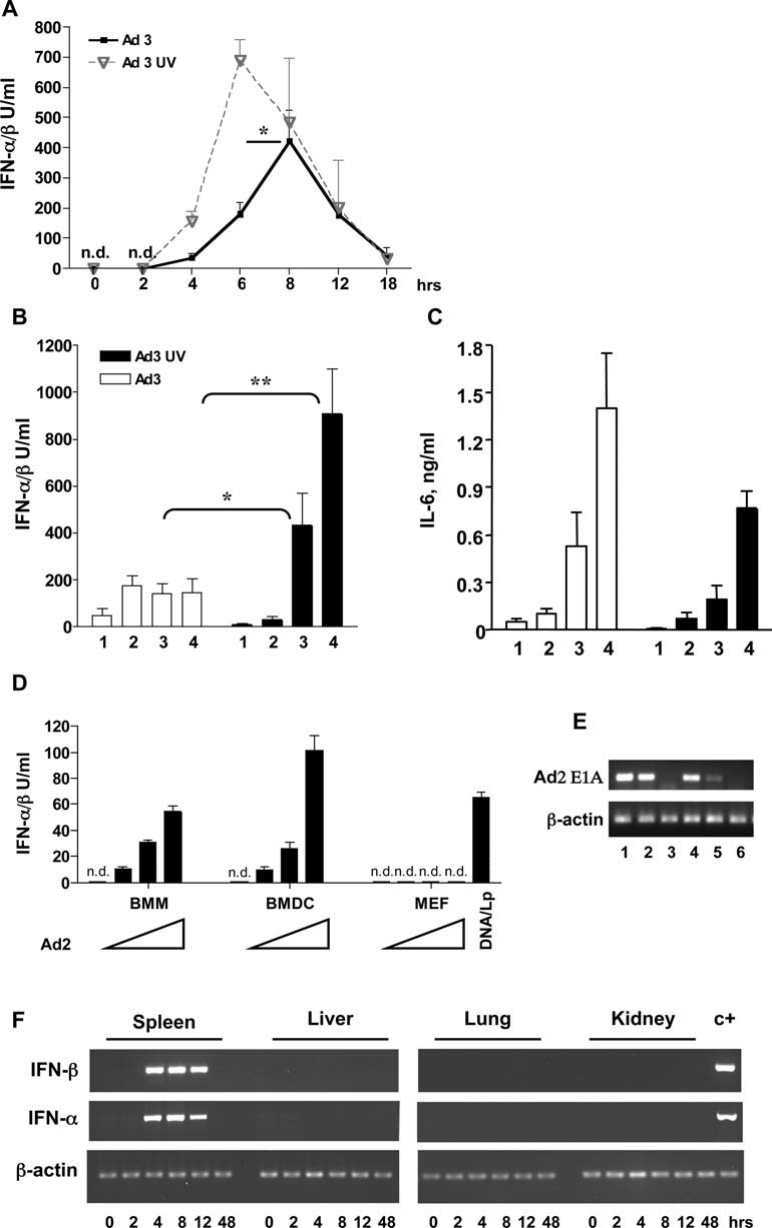
IFN-αβ and IL-6 responses to Ad infection *in vivo* and *in vitro*. (A) Kinetics of the IFN-αβ response. Mice (4–5/group) were infected intraperitoneally (i. p.) with 1.2×10^10^ intact Ad3 or 3.6×10^10^ of UV-inactivated Ad3 and IFN-αβ in plasma collected at the indicated time points was determined. 6 h and 8 h values were analyzed for statistical significance. One representative experiment of three is shown. (B, C) IFN-αβ and IL-6 responses to graded doses of Ad3. Mice (4–6/group) were infected with 4×10^9^ (1), 1.2×10^10^ (2), 3.6×10^10^ (3) or 7.2×10^10^ (4) of intact or UV-inactivated Ad3. Plasma for the determination of IFN-αβ and IL-6 was collected 6 h after infection. One representative experiment of two is shown. (D) Induction of IFN-αβ in different cell types by Ad in vitro. BMMs, BMDCs and MEFs were infected with 600, 1800 and 5400 Ad2 particles/cell or left uninfected. MEFs were also transfectected with Lipofectamine 2000-DNA complexes (DNA/Lp). IFN-αβ was measured in cell-free supernatants 6 h after infection. (E) Expression of viral E1A mRNA in infected cells. E1A and β-actin mRNAs were determined in cells 6 h after Ad2 infection by RT-PCR. Data in MEFs (lane 1, 2, 3) and BMDCs (lane 4, 5, 6) infected with 5400 (lane 1, 4) and 1800 (lane 2, 5) viral particles/cell and in mock infected cells (lane 3, 6) are shown. (F) Expression of IFN-β and IFN-α mRNA in organs of Ad-infected mice. (3 mice/group) were infected i.p. with 1.2×10^10^ Ad3 particles and the relative levels of IFN-β and IFN-α mRNA in organs were analyzed at the indicated time points by RT-PCR. A representative organ sample per each time point is shown. c+ indicates a spleen sample from a Ad3-infected mice used as a positive control. n.d.: not detectable.

In order to characterize the induction of type I IFNs by Ad *in vivo*, we first examined how two types of human Ad, Ad3 (species B) and Ad R700 (species C) [2] known to use distinct infectious entry routes, elicit an IFN-αβ response *in vivo*. The results summarized in Fig. 1A and supplementary Fig. S1A show that all mice infected with either of the two viruses exhibited similar IFN-αβ responses. IFN-αβ was first detectable in plasma at 4 h, peaking at 8 h and declining to low levels 18 h after infection. We then investigated, whether the expression of viral genes is required and/or regulate the induction of IFN-αβ by Ads. To this end, we injected mice with an UV-inactivated Ad3 (Fig. S1B) or Ad R700, incapable of viral gene expression, or with a recombinant Ad5 (species C) that contains a deletion of the E1 and E3 Ad early regions and expresses GFP (Ad5-GFP) (Fig. S1C). As shown in Fig. 1A, UV-inactivated Ads also induced a strong IFN-αβ response which, however, peaked at 6 h after injection, i.e. 2 h earlier than the response to intact Ads. A similar early-peaking IFN-αβ response was obtained in mice injected with UV inactivated Ad R700 (Fig. S2A) and with the recombinant Ad5-GFP (Fig. S2B).

Titration of the viral preparations in mice revealed that a positive correlation between the viral dose and the height of the IFN-αβ response existed only when relatively small doses of intact Ads were used. Higher doses of the Ads either did not elicit a further increase of the IFN-αβ response (Ad3, Fig. 1B) or led to a decrease of the response (Ad R700, Fig. S2C). In contrast, injection of the corresponding UV-inactivated Ads always led to a gradual increase of the IFN-αβ response and, at higher viral concentrations, even exceeded the response obtained with intact viruses. Thus, the expression of Ad genes is not required for the induction of IFN-αβ in mice. The data also indicate that the expression of early adenoviral genes negatively regulates type I IFN production. Notably, the Ad E1A gene has been shown previously to suppress Newcastle Disease Virus and IRF-3 induced IFN-α4 promoter induction in transient expression assays in fibroblasts [43].

Interestingly, however, viral gene expression did not inhibit the production of IL-6 in either Ad3- or Ad R700-infected mice (Fig. 1C and Fig. S2D). Further experiments revealed that, in contrast to the UV-inactivated Ad, heat-inactivated Ad did not elicit IFN-αβ in mice (data not shown). Since heat inactivation prevents the entry of Ad into cells ([44] and Fig. S2E), we conclude that signal transduction leading to IFN-αβ production is activated during Ad entry.

### Dendritic cells and macrophages, but not embryonic fibroblasts or L-929 cells, produce type I IFNs after Ad infection *in vitro*

Previous studies have shown that, depending on the inducing virus, IFN-αβ is produced ubiquitously or in a cell type specific manner [13],[14]. Here we stimulated different primary mouse cells, including bone marrow derived mDCs (BMDC), bone marrow derived macrophages (BMM), bone marrow derived pDCs and mouse embryonic fibroblasts (MEFs), with Ad2 (species C) or Ad3 (species B). Six hours later, the IFN-αβ content of culture supernatants and the expression of the viral E1A gene in the cells were determined. BMDC and BMM, but not MEFs, produced IFN-αβ (Fig. 1D), although all cell types were successfully infected as shown by RT-PCR (Fig. 1E and not shown). Very similar results were obtained when, instead of Ad2, Ad3 or the recombinant Ad5-GFP were used for infection of the three cell types (not shown). In addition, pDCs also produced IFN-αβ in response to Ad infection in vitro (Fig. S3A); however, only at high multiplicities of infection. Finally, like MEFs, L-929 cells infected with Ad2 produced no IFN-αβ either (Fig. S3B). In agreement with previous studies [36],[37], MEFs and L-929 cells produced IFN-αβ following transfection with purified DNA (Fig. 1D and Fig. S10C). In addition to mouse DCs and macrophages we also found that human monocyte-derived DCs produced IFN-αβ upon infection with the adenoviral vector Ad5-GFP (Fig. S3C). The present data confirm that Ads can trigger IFN-αβ production in various immune cells in vitro [10],[45],[46]. However, it furthermore indicates that production does not proceed ubiquitously in all types of infected cells.

### Splenic myeloid dendritic cells are the major producers of IFN-αβ in Ad-infected mice

In order to identify the organ site of viral uptake and IFN-αβ production *in vivo*, we analyzed the expression of the early viral gene E1A and of type I IFN mRNAs, respectively, in the spleen, liver, lung and kidney of Ad3-infected mice. We found expression of the early viral E1A gene in spleen and liver (Fig. S1B), but not in lung and kidney (not shown), which agrees with earlier findings on *in vivo* Ad tropism [47],[48]. Expression of IFN-α and IFN-β mRNA was below the level of detection in the organs of non-infected controls. We also found that between 4 and 18 h after infection IFN-α and IFN-β mRNAs were expressed at high levels in the spleen (Fig. 1F), but surprisingly not in the liver, despite the presence of Ad in both organs. As expected, IFN-αβ was not expressed in the virus-free lung or kidney of infected mice (Fig. 1F).

**Figure 2:**
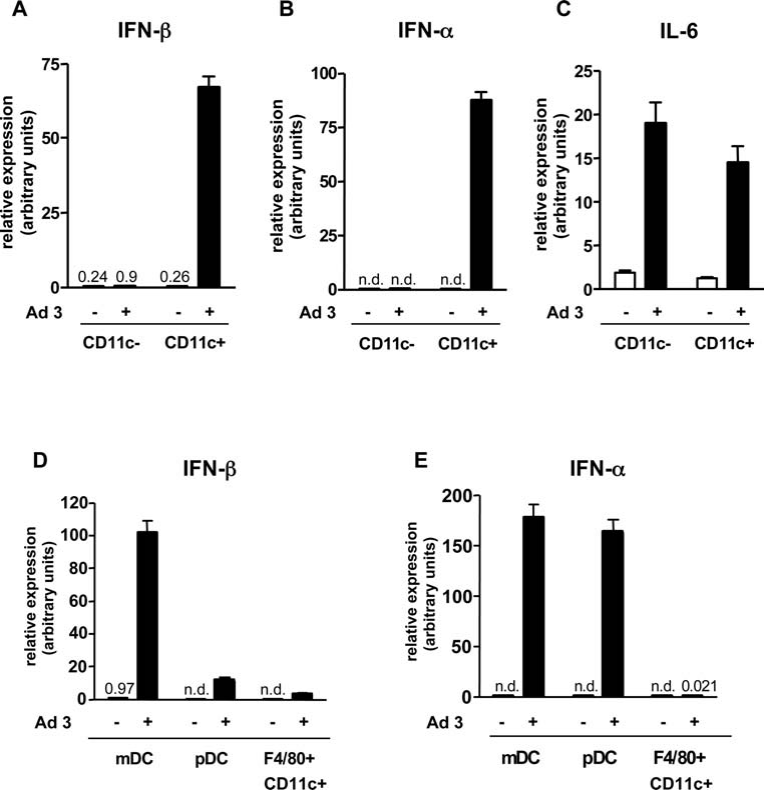
IFN-αβ and IL-6 mRNA levels in *ex vivo* purified splenocyte subsets from Ad infected mice. Spleens were removed from mice 8 hours after infection with 1.2×10^10^ Ad3 particles and from uninfected mice. Pooled splenocytes (3–5 mice/group) were MACS enriched for CD11c^+^ and CD11c^−^ cells and further purified with FACS-sorting after staining with antibodies to CD11c, Cd11b, Gr-1, B220 and F4/80. Gates used for the purification of DC subsets are shown on Figure S4. The relative expression of IFN-β (A, D), IFN-α (B, E) and IL-6 (C) was measured in CD11c^+^ versus CD11c^−^ cells (A,B,C) and in the mDC, pDC and F4/80^+^CD11b^−^ subsets of CD11c^+^ cells (D, E) using real-time RT-PCR. Triplicate measurements of a representative experiment of three are shown. n.d.: not detectable.

In order to identify the cell type(s) producing IFN-αβ *in vivo*, we isolated splenocytes from Ad infected animals 8 h after virus treatment and separated them into CD11c^+^ (DC-containing) and CD11c^−^ (non-DC) populations. Both CD11c^+^ and CD11c^−^ populations contained viral DNA (not shown). This finding is in accordance with the report of Morelli et al describing that both splenic DC and non-DC contain the virus in Ad-infected mice [49]. However, quantitative RT-PCR determination of IFN-α and IFN-β mRNA in both populations revealed the presence of IFN-αβ mRNAs predominantly in the DC-containing CD11c^+^ fraction (Fig. 2A, B), but not in the macrophage containing CD11c^−^ fraction. In contrast to IFN-α and IFN-β, the mRNA levels of IL-6, another cytokine known to be induced by Ad, were comparably upregulated in both the CD11c^+^ and CD11c^−^ population (Fig. 2C). Since the latter non-DC population comprises more than 95% of all mouse splenocytes [50], we conclude that most of the IL-6 made in the Ad-infected spleen is of non-DC origin.

**Figure 3:**
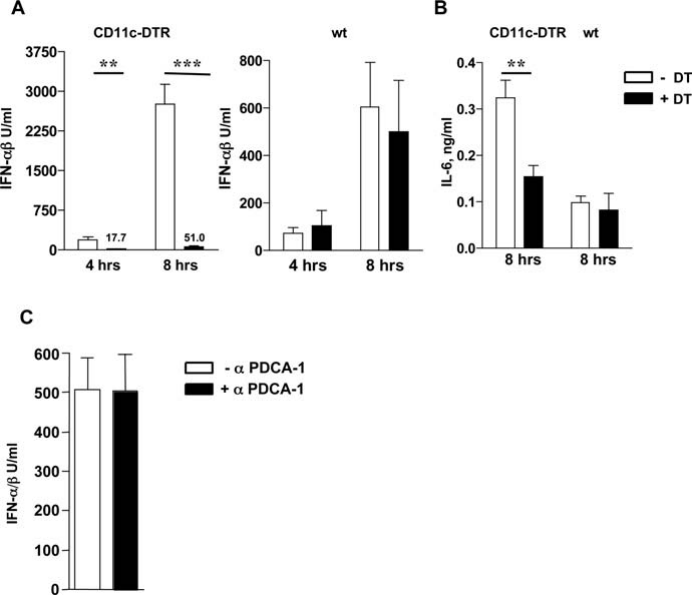
Splenic mDCs are the major IFN-αβ-, but not the predominant IL-6-producing cells in adenovirus infected mice. Ad-induced IFN-αβ and IL-6 responses in mice depleted for mDCs and pDCs. Groups of wt and CD11c-DTR mice (4/group) were injected with DT i. p. or left untreated. 24 h later the animals were injected with 1.2×10^10^ Ad3 particles and the plasma levels of IFN-αβ (A) and IL-6 (B) were measured 4 and 8 h after infection. Mice (4/group) were untreated or injected with 500 mg of anti-mPDCA-1 antibody i. p. and challenged with 1.2×10^10^ Ad3 particles. Plasma IFN-αβ levels were determined 8 h after Ad infection (C). The efficacy of splenic DC depletions is shown in Fig. S5 and S6.

According to published data, the CD11c^+^ cell population contains different types of DCs, as well as other cells such as lymphocytes, macrophages, granulocytes and NK cells [51],[52],[53]. We therefore further separated the purified CD11c^+^ cells into mDCs (CD11c^+^CD11b^+^Gr1^−^), pDCs (CD11c^+^CD11b^−^GR1^+^B220^+^) and a CD11c^+^CD11b-F4/80^+^ subpopulation (Fig. S4A–D) and measured the expression of IFN-α, IFN-β and β-actin with real-time RT-PCR. After normalization to β-actin expression, we found that on a per cell basis mDCs expressed significantly more IFN-β than pDCs (Fig. 2D), but both mDCs and pDCs expressed similar amounts of IFN-α (Fig. 2E). In contrast, CD11c^+^, CD11b^−^ cells carrying the macrophage marker F4/80^+^ did not express detectable amounts of IFN-α or IFN-β. Since mDCs comprise approximately 60% of all analyzed CD11c^+^ splenocytes and their numbers are approximately 10-times higher than those of pDCs (Fig. S5A, B and [50]), these results suggested that the vast majority of IFN-αβ in Ad-infected mice was produced by splenic mDCs. To verify this assumption, we analyzed the Ad-elicited cytokine responses in mice depleted of CD11c^high^ MHC II^+^ myeloid DCs. To ablate these cells, we injected diphtheria toxin into the CD11c-diphtheria toxin receptor CD11cDTR/GFP transgenic mice [54] 24 h prior to infection with Ad. In agreement with previous reports [55],[56], DT pre-treatment of DTR/GFP transgenic mice resulted in the ablation of CD11c^high^ MHC II^+^ CD11b^+^ splenic mDCs, whereas the CD11c^int^ Siglec H^+^ CD11b^−^ plasmacytoid DCs remained unaffected (Fig. S5A, B). When DT pre-treated CD11cDTR/GFP transgenic mice were challenged with Ad3 and examined for IFN-αβ in plasma 4 and 8 h after infection, only marginal IFN responses were found at both time-points, in contrast to the strong responses of similarly infected transgenic control mice that had not received DT (Fig. 3A). The same pretreatment with DT had no effect on the Ad elicited IFN-αβ response of wild-type mice (Fig. 3A).

Interestingly, the determination of IL-6 levels in plasma 8 h after infection revealed that the ablation of mDCs in CD11cDTR/GFP transgenic mice affected only moderately the induction of IL-6 (Fig. 3B), confirming that non-DCs contribute significantly to the Ad-elicited IL-6 response *in vivo*. The fact that different cell types are responsible for IFN-αβ and IL-6 response to Ad may explain at least in part why viral gene expression did not inhibit the production of IL-6 in Ad3- or Ad R700-infected mice (see Fig. 1C and Fig. S2D). In order to functionally evaluate the possible participation of pDCs to the systemic production of IFN-αβ to Ad we also checked this response in mice depleted of pDCs. As shown in Fig. S6A and B, the injection of anti PDCA-1 antibody led to the substantial decrease of the number of splenic pDCs. Nevertheless, the production of IFN-αβ was not changed in response to Ad infection (Fig. 3C). The data collectively show that the vast majority of IFN-αβ but not of IL-6 in Ad infected-mice is produced by splenic mDCs.

### Ad induction of type I IFNs is TLR independent

**Figure 4:**
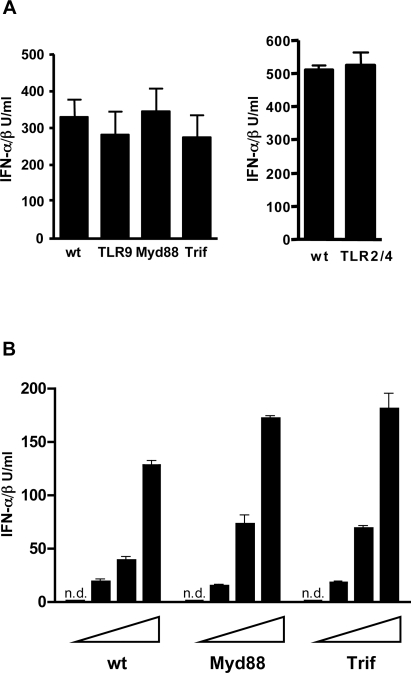
Adenovirus induction of IFN-αβ is TLR independent. (A) IFN-αβ responses to Ad in mice with impaired TLR signaling. IFN-αβ was measured in plasma of mice (4–6/group) of the indicated genotypes 8 h after i. p. infection with 1.2×10^10^ Ad3 particles. One representative experiment of three is shown. (B) IFN-αβ response to Ad in BMDC with impaired TLR signaling. BMDC generated from wt, Myd88^−/−^ and TRIF^−/−^ mice were infected *in vitro* with 600, 1800 and 5400 Ad3 particles/cell or were mock-infected. IFN-αβ was measured in cell-free supernatants 16 h after infection. One representative experiment of three is shown. n.d.: not detectable.

Recent studies have shown the involvement of different TLRs including TLR 2, 3, 4, 7 and 9 in the innate recognition of different viruses [16],[17],[18],[57],[58],[59],[60],[61]. Signaling via TLR9 was shown to be responsible for the strong type I IFN response of pDCs to Ad *in vitro* [10],[45],[46]. Here we investigated the possible involvement of TLR9 in the induction of type I IFNs by measuring IFN-αβ in Ad infected wt and TLR9^−/−^ mice. Fig. 4A shows that TLR9^−/−^ mice produced normal levels of IFN-αβ. upon infection with Ad3. Comparable results were obtained with the recombinant Ad5-GFP (Fig. S7A). Moreover, Unc93B mice, deficient in signalling by intracellular TLRs showed normal IFN-αβ responses to Ad5-GFP (Fig. S7A). We further checked the possible involvement of the TLR system using mice deficient for TLR2, TLR4 or for the TLR adaptor proteins TRIF and MyD88. Fig. 4A shows that the Ad3 induced IFN-αβ levels in all strains of mice were as high as in the respective wt controls. Similarly, comparable IFN-αβ responses were also found in TLR-, MyD88- or TRIF- deficient mice and in the corresponding wt animals after infection with UV-inactivated Ad3, AdR700 and Ad5-GFP (not shown). Furthermore, comparable IFN-αβ responses were found in cultures of Ad-infected BMDCs from wt, MyD88- and TRIF-deficient mice (Fig. 4B). The various TLR deficient mice showed impaired responses to the corresponding ligands in control experiments (Fig. S7B). Collectively, our data show that the TLR system plays no major role in the systemic IFN-αβ responses to Ads in mice.

### The induction of IFN-αβ and IL-6 in response to Ad infection is dependent on IFN-αβ feedback signaling

**Figure 5:**
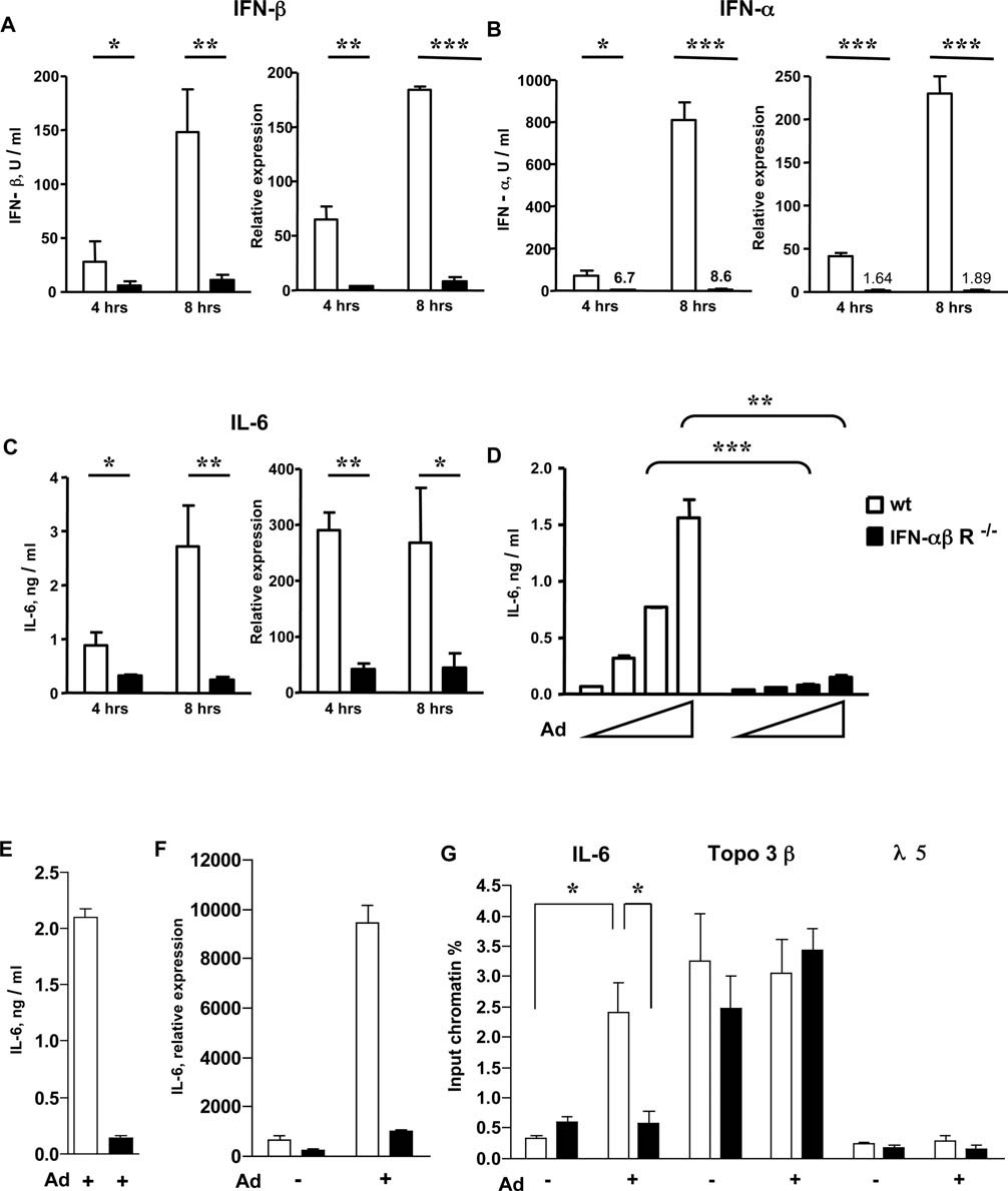
Role of IFN-αβ feedback in Ad-induced IFN-αβ and IL-6 responses. (A–C) Role of IFN-αβ feedback signaling *in vivo*. Wt (empty bars) and IFN-αβR^−/−^ (black bars) mice (4–6/group) were infected with 6.2×10^10^ Ad3 particles. Four and 8 h after infection, the protein and mRNA levels of IFN-β (A) IFN-α (B) and IL-6 (C) were determined in plasma and spleen with bioassay and real-time RT-PCR, respectively. (D-G) Role of IFN-αβ feedback signaling in Ad induced IL-6 production. BMDCs (D) from wt and IFNαβR^−/−^ mice were mock-infected or infected with 600, 1800 and 5400 Ad3 particles/cell. Wt and IFNαβR^−/−^ BMMs were mock-infected or infected with 5400 Ad5 GFP particles/cell (E–G). IL6 was measured in cell-free supernatants 16 h (D) or 8 h (E) after infection. IL-6 mRNA was quantified 8 h after infection (F). Acetylation of histone H4 was measured at the IL-6, topoisomerase 3β and λ5 promoters 8 h after infection. Mock immunoprecipitated samples showed no significant values (less than 0.1%) for all samples, not shown. (G). One representative experiment of three (D) or two (E) is shown.

The type of virus, the IFN-αβ-producing target cell, and the activation mechanism determines whether positive feedback signaling is involved in the induction of the IFN-αβ response or not [13],[20]. Here, we studied the possible involvement of IFN feedback signaling in the IFN-αβ and IL-6 response to Ad3 by using wt and IFN-αβR-deficient mice. The absence of the IFN-αβ receptor resulted in dramatically decreased levels of IFN-α and IFN-β protein in the plasma as well as of IFN-α and IFN-β mRNAs in the spleen (Fig. 5A, B) 4 and 8 h after Ad infection. The difference between the protein or mRNA levels of IFN-α and IFN-β in wt versus mutant animals was approximately 100 and 20-fold, respectively. Furthermore, in contrast to wt mice, the characteristic rise in IFN-αβ levels between 4 and 8 h after infection was absent in IFN-αβR^−/−^ mice. Thus, in Ad-infected mice, production of both IFN-α and IFN-β is strictly dependent on positive IFN-αβ feedback. The determination of IL-6 protein and mRNA levels in the same plasma and splenic tissue samples revealed that the induction of IL-6 is also positively regulated by IFN-αβ signaling (Fig. 5C), which is in agreement with a previous study [10].

We also tested whether IFN-αβR-dependent signaling is involved in the IFN-αβ response of Ad-infected BMDCs *in vitro*. As with the *in vivo* results, we found that cells from IFN-αβR^−/−^ mice infected with Ad3 produced significantly less type I IFN than similarly infected cells from wt mice (Fig. S8). The loss of IFN-αβ signaling also resulted in a strong inhibition of the Ad induced IL-6 production in BMDCs (Fig. 5D) and BMMs (Fig. 5E). Furthermore, as shown using Ad-infected BMMs, it resulted also in a strong reduction of inducibility of IL-6 mRNA expression (Fig. 5F). Because transcriptional changes are often determined by epigenetic factors [62] we checked the levels of hyperacetylated histone H4 (acH4), a permissive factor for transcription at the IL-6 promoter in control and Ad-infected BMMs from wt and IFNαβR^−/−^ mice. Chromatin immunoprecipitation (ChIP) assays showed a significant enrichment of acH4 at the IL-6 promoter in infected wt, but not IFNαβR^−/−^ BMMs (Fig. 5G). In a control experiment, as expected, an enrichment of acH4 was observed at the promoter of the constitutively active Topoisomerase 3β but not of the l5 (not expressing, active only in early B-cells) gene in both cells types. From these results we conclude that IFN-αβ exerts a positive regulatory effect on the Ad-induced IL-6 transcription and that its loss is at least partly responsible for the strong reduction of the IL-6 response in Ad-infected IFN-αβR knockout mice.

**Figure 6:**
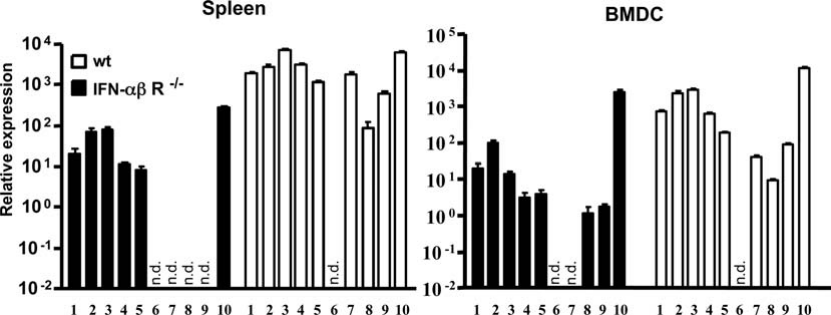
Ad-induced pattern of IFN-αβ subtypes *in vivo* and *in vitro* in the presence and absence of IFN-αβ feedback. The expression of different IFN-αβ subtypes was analyzed by real-time RT PCR in total RNA prepared from the spleen or from *in vitro* generated BMDCs of wt or IFN-αβR^−/−^ mice 8 h after infection with 6.2×10^10^ Ad3 particles/mouse (*in vivo*) or with 900 particles/cell (*in vitro*). One representative experiment of two is shown. (1) IFN-α2, (2) IFN-α4, (3) IFN-α5, (4) IFN-α6, (5) IFN-α9, (6) IFN-α11, (7) IFN-α12, (8) IFN-α13, (9) IFN-α14, (10) IFN-β. n.d.: not detectable.

Using real-time RT-PCR we then analyzed the spectrum of IFN-αβ genes in wt mice as well as the impact of IFNαβR deficiency on their induction. Included were IFN-α2, 4, 5, 6, 9, 11, 12, 13, 14 and IFN-β. All of them were induced in the spleen by Ad3 *in vivo* and in BMDC *in vitro*. IFN-αβ subtypes were not detectable in the spleen of unstimulated mice or BMDCs (not shown). Fig. 6 shows the patterns of IFN-αβ genes induced *in vivo* and *in vitro* in the presence or in the absence of IFN-αβ feedback signaling. In wt mice and cells IFN-α5 and IFN-β were the most strongly expressed genes and IFN-α13 was the least activated IFN-α subtype. IFN-α11 was not induced at all. IFNαβR deficiency resulted in an inhibition of IFN-αβ gene expression, the strength of which was subtype dependent. In some cases the inhibition was weak (IFN-α2, 4, 5 and IFN-β), in others strong or complete (IFN-α12, 13, and 14), showing that the expression of different subtypes of IFN-αβ are differentially affected by IFN-αβ feedback signaling. Collectively, these data show that the adenovirus triggered production of IFN-αβ and IL-6 in BMDCs *in vitro* and in mice *in vivo* is strongly dependent on intact IFN-αβ signaling.

### The critical role of IRF-7 but not IRF-3 in Ad-induced type I IFN production

**Figure 7:**
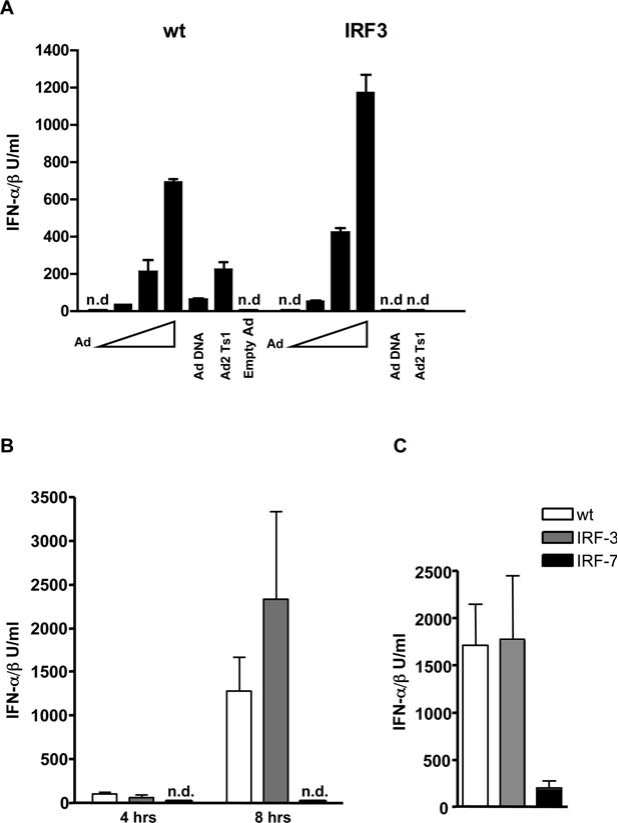
Adenovirus induction of IFN-αβ requires IRF-7 but is independent on IRF-3. Wt and IRF-3^−/−^ BMDCs were infected with 0, 600, 1800 and 5400 Ad5GFP particles/cell or were transfected with Ad5GFP DNA (2 µg/ml), Ad2Ts1 (2×10^9^ whole virus particles/ml) and empty Ad5 GFP (2×10^9^ whole virus particles/ml) with Lipofectamine 2000. IFN-αβ was measured in cell-free supernatants 16 h after infection (A). One representative experiments of three is shown. Wt, IRF-3^−/−^ and IRF-7^−/−^ mice (4–6/group) were infected with 6.2×10^10^ Ad3 particles, i.p. (B) or injected with 5 µg of poly I∶C i. p. (C) Plasma IFN-αβ was measured 4 and 8 h (B) or 3 h (C) after stimulation. One representative experiment of two is shown. n.d.: not detectable.

The transcription factors IRF-3 and IRF-7 have distinct and important roles in IFN-αβ production induced by viruses or other pathogens and their involvement can be characteristic for the induction mechanisms involved [13]. Specifically, IRF-3 has been shown to be critically involved in cytoplasmic DNA sensing and in the Ad-induced IFN-αβ production in BMMs *in vitro* [63]. We show here that IRF-3 is critical for the induction of IFN-αβ by isolated adenoviral DNA, but not by infection with whole virions in BMDCs (Fig. 7A). In order to analyze the individual contribution of IRF-3 and IRF-7 to the Ad-induced IFN-αβ response *in vivo*, we infected mice deficient for these transcription factors with Ad3. We also compared these responses to those triggered by poly I∶C, a known activator of the cytosolic IFN-αβ producing pathway *in vivo*. As shown in Fig. 7B, the lack of IRF-3 did not significantly influence the plasma levels of IFNαβ 4 or 8 h after infection in response to Ad. In contrast, Ad-infected IRF-7-deficient mice did not exhibit detectable amounts of IFN-αβ in the plasma. Very similar data were obtained with Ad5GFP (Fig. S9). Thus, IRF-7, but not IRF-3, is essential for the induction of the IFN-αβ response during Ad infection. Compared to wt mice, poly I∶C injected IRF-7 deficient mice produced significantly less, but still well detectable amounts of IFN-αβ.

### The nucleic acid sensors MDA-5, RIG-I and DAI/Zbp1 are not critical for Ad-induced type I IFN production

**Figure 8:**
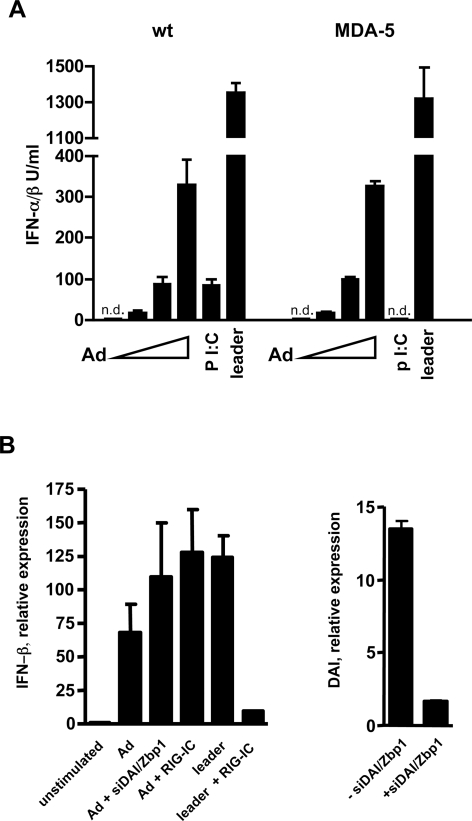
IFN-αβ production after adenovirus induction is independent of known cytosolic nucleic acid sensors. Wt and MDA-5^−/−^ BMDCs were infected with 0, 600, 1800 and 5400 Ad5GFP particles/cell, or stimulated with synthetic poly I∶C (10 µg/ml) or leader RNA complexed with Lipofectamine 2000 (0.1 µg/ml). IFN-αβ was measured in cell-free supernatants 16 h after infection (A). IRF-3^−/−^ BMDCs were transfected with pmaxGFP (1 µg/3×10^5^ cells) alone and together with RIG-IC (1 µg/3×10^5^ cells) or with DAI/Zbp1 targeting siRNAs (0.3 µM/3×10^5^ cells). Twentyfour hours later cells were infected with 5400 Ad5GFP particles/cell. Alternatively, RIG-IC transfected cells were stimulated with synthetic leader RNA (0.1 µg/ml) complexed with Lipofectamine 2000. Expression of IFN-β mRNA was measured in sorted GFP^+^ cells 12 h after stimulation. Knockdown of DAI/Zbp1 expression was measured in siRNA transfected cells (B).

Next, we investigated whether the cytoplasmic RNA sensors MDA-5 or RIG-I, or the putative DNA sensor DAI/Zbp1 may play a major role in the induction of the IFN-αβ response to Ad. The possible involvement of MDA-5 was tested using BMDCs and BMMs from MDA-5 deficient mice. Normal IFN-αβ responses to Ad were obtained in MDA-5-deficient BMDCs cells (Fig. 8A) and also in BMMs (not shown), whereas the responses to the known MDA-5 ligand Poly I∶C were abrogated (Fig. 8A). The possible involvement of RIG-I was checked using a dominant negative form of RIG-I (RIG-IC) [64]. BMDCs from IRF-3^−/−^ mice were transfected with a GFP-expressing plasmid (transfection control) with or without RIG-IC and subsequently stimulated with Ad or control leader RNA. We used IRF-3^−/−^ cells to avoid any induction of IFN-αβ by the plasmid itself, which is, contrary to that by Ad, strictly IRF-3 dependent. The induction of IFN-β mRNA was analyzed in sorted GFP-positive cells. As shown in Fig. 8B, left, the transfection of RIG-IC prevented induction of IFN-β mRNA by the leader RNA, but not by Ad. The role of Zbp1/DAI was analyzed using siRNA-mediated knockdown of DAI/Zbp1 in BMDCs. For this purpose, cells from IRF-3−/− mice were co-transfected with DAI/Zbp1 targeting siRNAs and a GFP expressing plasmid and subsequently stimulated with Ad. As shown in Fig. 8B, right, the transfection of BMDCs resulted in strongly reduced DAI/Zbp1 expression but not in a reduced IFN-β mRNA induction by Ad in GFP-positive cells. Control experiments with L-929 cells showed that transfection of the gene-specific siRNA downregulated the expression of DAI/Zbp1 on both the mRNA and protein levels and efficiently inhibited the IFN-β response to B-DNA (Fig. S10A–C). Collectively, our results indicate that known nucleic acid sensors such as MDA-5, RIG-I and Zbp1/DAI are not involved in the Ad-induced type I IFN production.

### SAPK/JNK signaling is activated upon Ad infection and is necessary for virus elicited IFN-αβ and IL-6 production

**Figure 9:**
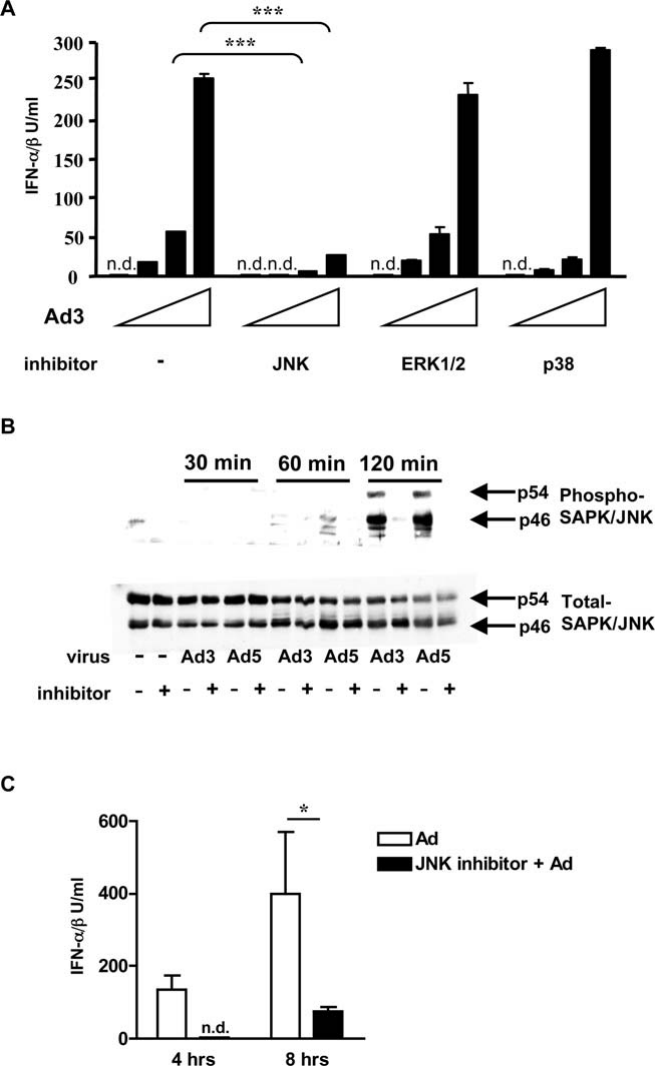
Adenovirus induction of IFN-αβ is dependent on SAPK/JNK signaling. (A) Effect of MAPK inhibitors on the IFN-αβ response of BMDC to Ad. BMDC from mice were pretreated for 15 min with the inhibitor SP600125 (for SAPK//JNK), UO126 (for ERK1/2), SB 203580 (for p38) or with the diluent only as described in Materials and Methods, and then infected with 600, 1800 and 5400 Ad3 particles/cell or mock-infected. IFN-αβ was measured in cell-free supernatants 6 h after infection. n. d.: not detectable. (B) Inhibition of SAPK/JNK activation by SP600125 in Ad-infected BMDC. BMDCs were pretreated with SP600125) or with diluent for 15 min and then infected with 5400 Ad3 or Ad5 particles/cell, or were mock-infected. Total cell lysates obtained at the indicated times after Ad were analyzed by immunoblotting using antibodies to the total or phosphorylated p46 and p54 SAPK/JNK isoforms. (C) The effect of SP600125) treatment on the IFN-αβ production in Ad-infected mice. *B6* Mice (4/group) were pretreated with the SP600125 inhibitor (20 mg/kg) i.p. or with diluent for 45 min and then infected with 3.6×10^10^ Ad5-GFP particles. Plasma IFN-αβ was measured 4 and 8 h after infection. One representative experiment of three is shown.

MAPKs have been previously shown to be activated by Ad *in vitro*, in different non-immune cell types and to be important for the induction of chemokines in response to Ad [65],[66],[67],[68]. Here we investigated whether members of the MAPK family play a role in the Ad-induced IFN-αβ and IL-6 response. We infected BMDCs with Ad3 in the presence or absence of MAPK inhibitors and determined the levels of IFN-αβ and IL-6 produced. Fig. 9A and Fig. S11 show that the pharmacological blockade of the SAPK/JNK MAPK almost completely inhibited the Ad3-induced production of both IFN-αβ and IL-6. In contrast, the inhibition of the p38 MAPK pathway partially inhibited the production of IL-6, but had no effect on the production of IFN-αβ. Finally, the blockade of ERK1/2 had no effect on the production of either IL-6 or IFN-αβ. Very similar data on the effects of MAPK inhibitors were obtained using Ad2 and mutant Ad5-GFP to stimulate BMDC (data not shown).

Next, we analyzed the levels of activated SAPK/JNK MAPK proteins in BMDCs and found their robust phosphorylation 2 h after either Ad3 or Ad5-GFP infection (Fig. 9B). We also tested the importance of SAPK/JNK signaling on Ad-induced IFN-αβ production *in vivo*. Fig. 9C shows that the blockade of the SAPK/JNK signaling pathway in mice completely inhibited the production of IFN-αβ at 4 and partially at 8 h after Ad infection. Taken together, these data strongly indicate that the Ad-activated SAPK/JNK MAPK pathway plays an important role in the virus-induced production of type I IFNs and IL-6.

### Endosomal escape triggers the Ad-induced production of IFN-αβ and IL-6, but prevents TLR9-dependent innate recognition

**Figure 10:**
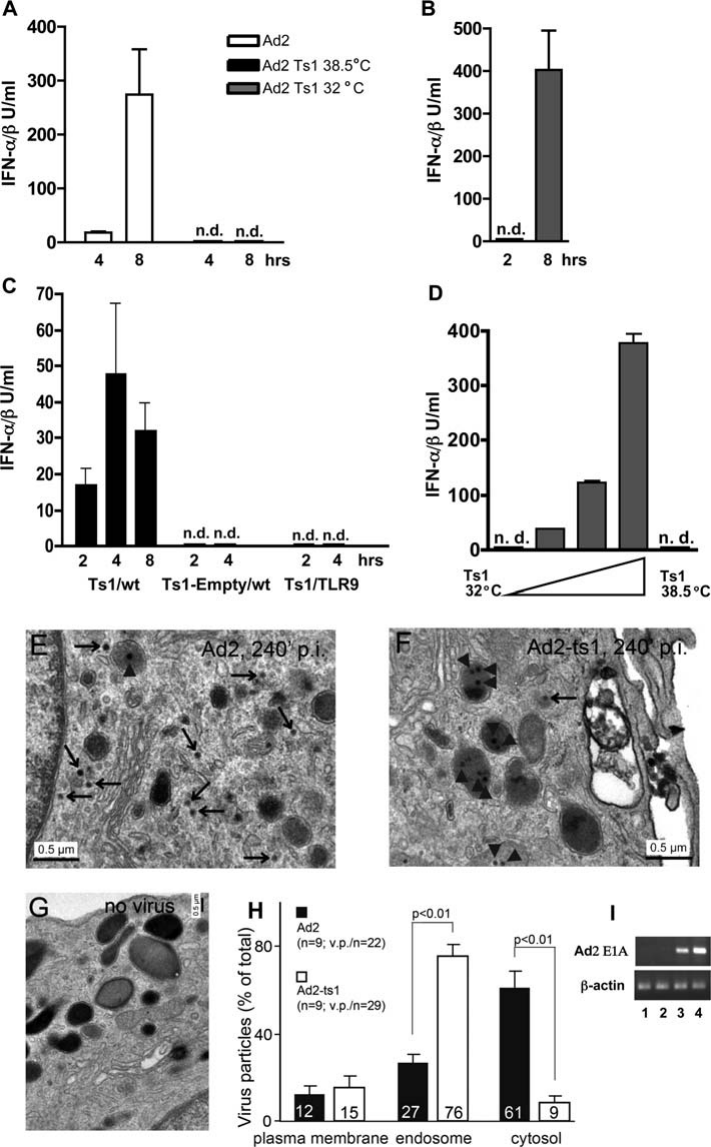
Requirement for viral endosomal escape in the induction of IFN-αβ by adenovirus *in vivo* and *in vitro*. Comparison of the cytokine responses of mice infected with wild-type Ad2 or mutant Ad2Ts1 virus grown at the non-permissive (38.5°C) or permissive (32°C) temperature. B6 Mice (4/group) were infected with 3.6×10^10^ virus particles i. p. and plasma IFN-αβ was measured 4 and 8 h (A) or 2 and 8 h (B) after infection. n.d.: not detectable. IFN-αβ responses to high doses of Ad2Ts1 virus *in vivo*. Wt or TLR9 deficient (4 animals/group) mice were infected with 2.16×10^11^ Ad2Ts1-38.5°C particles, i.p. or with the same number of Ad2Ts1-38.5°C empty particles as indicated. IFN-αβ levels were measured in plasma at the indicated time-points after infection (C). Comparison of IFN-αβ responses of BMDCs infected with Ad2Ts1 grown at permissive or non-permissive temperature. BMDCs were infected with 1800, 5400, and 16 200 particles of Ad2Ts1-32°C/cell or with 32 400 particles of Ad2Ts1-38.5°C/cell or were mock-infected. IFN-αβ was measured in cell-free supernatants 16 h after infection (D). One representative experiment of two is shown. (E–H) Incoming Ad2 but not Ad2-ts1 particles localize to the cytosol of mDCs. Myeloid dendritic cells were infected with Ad2 or Ad2-ts1 (38.5°C), warmed to 37°C for 4 h, fixed and processed for transmission EM analysis as described in Materials and Methods. (E) and (F) show images of Ad2 and Ad2-ts1 infected cells, respectively, and panel (G) depicts a fraction of a non-infected cell. Statistical analyses of Ad2 and Ad-ts1 particles at the plasma membrane outside of the cell, in endosomes and the cytosol are shown in (H). (I) Absence of viral *E1A* expression in the spleen of Ad2Ts1-infected mice. Mice (2/group) were infected with 3.6×10^10^ Ad2 or Ad2Ts1 (Ts1) particles, i. p. The expression of E1A mRNA in the spleen 6 h after infection was analyzed by RT-PCR in samples from the individual Ts1-infected (lane 1, 2) and Ad2-infected (lane 3, 4) mice.

During the course of our adenovirus preparations, we regularly found “empty capsids” which we separated and purified in addition to the mature virions. We tested the IFN-αβ stimulating activity of these preparations *in vivo* and observed that they were not active (Fig. S12A). Since empty capsids lack viral DNA and exhibit an altered protein composition [69], the absence of an inducing viral constituent(s) from these capsids could explain their inability to provoke an IFN-αβ response. Another possible explanation could be that endosomal escape is required for IFN-αβ induction, since empty capsids cannot escape from the endosome [69]. To test the latter possibility, we infected mice with 3.6×10^10^ viral particles of wt Ad2 and Ad2Ts1, a viral mutant deficient in endosomal escape [5]. As shown in Fig. 10A, in contrast to the wt virus, Ad2Ts1 did not induce detectable levels of type I IFN at 4 and 8 h after infection. Furthermore, the IL-6 response was also severely reduced in these animals (Fig. S12B). In a control experiment, mice infected with Ad2Ts1 grown at permissive temperature (32°C) and thus capable of endosomal escape, exhibited normal IFN-αβ responses (Fig. 10B). The inability of Ad2Ts1 to escape from endosomes of mDCs was confirmed by electron microscopy (Fig. 10E–H) and *in vivo* by the lack of Ad early E1A gene expression in the spleen of mice infected with Ad2Ts1 (Fig. 10I). Thus, escape from the endosome is critical for the induction of IFN-αβ and IL-6 by adenoviruses.

It should be noted that a further increase in the Ad2Ts1 dose (2.16×10^11^ particles) resulted in detectable, albeit very low levels of plasma IFN-αβ that were released with different kinetics (Fig. 10C). In this case, IFN-αβ was detectable as early as 2 h after infection and, in contrast to the results we obtained with wt viruses (Figures 1A, 10A and S1A), the levels of IFN-αβ did not increase significantly at the later time-points. This already suggests a mechanism for type I IFN induction by Ad2Ts1 that is fundamentally different from the IFN-αβ induction seen with wt Ad2. Since Ads are DNA viruses, they can possibly be detected by TLR9. In fact, the innate immune recognition of Ad in pDCs is TLR9 dependent. We therefore repeated the experiment with a high dose of Ad2Ts1 using TLR9^−/−^ mice. As shown in Fig. 10C, TLR9^−/−^ mice did not produce IFN-αβ in response to the mutant Ad2Ts1, quite in contrast to the results obtained with the wt virus (see Fig. 4A) Likewise, there was no detectable IFN-αβ release in Ad2Ts1-infected mice deficient in MyD88, an essential component of TLR9 signaling (not shown). Furthermore, the corresponding amount of empty particles (DNA-free) of Ad2Ts1 elicited no IFN-αβ response in wt mice (Fig. 10C). These data illustrate the critical role of TLR9 in the induction of IFN-αβ by means of high doses of Ad2Ts1. To exclude the possibility that contaminating DNA on the surface of the virions was responsible for the TLR9-dependent IFN-αβ induction, we treated the mutant virions with bensonase, which destroys all kinds of free nucleic acids. Bensonase-treated Ad2Ts1 still induced an IFN-αβ response in mice (not shown) supporting the view that endogenous viral and not contaminating DNA is responsible for IFN-αβ induction by the Ad trapped in the endosomes.

We also tested the role of endosomal escape of Ad in the *in vitro* induction in BMDCs of IFN-αβ and IL-6. Fig. 10D shows a dose dependent production of IFN-αβ by Ad2Ts1 grown at permissive temperature and Figures S12C and D the production of IFN-αβ and IL-6 by wt Ad2, but no production of either of the cytokines by Ad2Ts1 grown at the restrictive temperature. Interestingly, liposomal transfection of whole Ad2 Ts1 virions (but not of empty virus particles) in BMDCs resulted in the significant production of IFN-αβ that however, was critically dependent on IRF-3 (Fig. 7A). Similarly, Ad2 Ts1 did not induce IFN-αβ production in human monocyte derived DCs (Fig. S12E). The requirement of low pH for Ad3 infection was also tested using bafilomycin A1, a drug known to inhibit the acidification of endosomes. Experiments shown in Fig. S13A, B revealed that cells treated with this drug produced significantly reduced levels of IFN-αβ and IL-6 respectively, in response to Ad3. This is consistent with the notion that Ad3 and Ad7 infection of cultured cells requires low endosomal pH [70],[71]. Similar results were obtained using treatment with ammonium chloride, another acidification inhibitor known to block Ad escape from endosomes (not shown). Collectively, the data *in vitro* and *in vivo* provide evidence that the late phase of the Ad infectious entry, in which the virus escapes from the endosome, triggers an innate response characterized by the production of type I IFNs and IL-6.

### Ad elicited IFN-αβ production leads to LPS hypersensitivity

**Figure 11:**
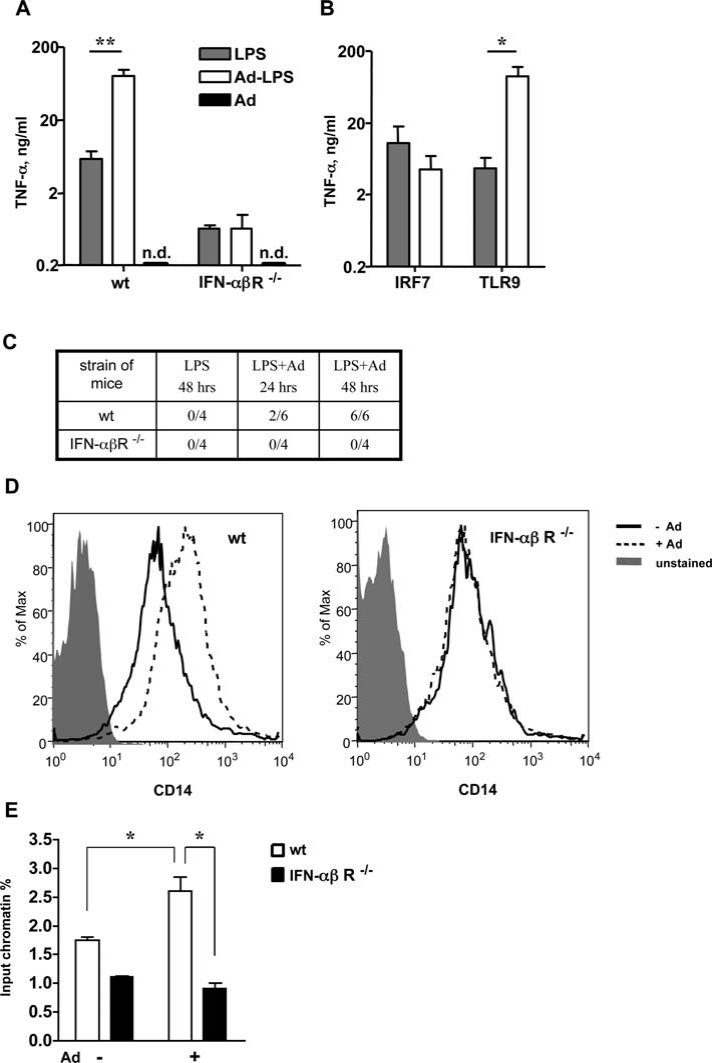
Adenovirus triggered IFN-αβ production is the mediator of LPS hypersensitivity. Wild-type and IFN-αβR^−/−^ mice (4–6 mice/group) were infected i. p. with 1×10^10^ Ad3 particles or left uninfected. 16 h later the animals received 1 µg LPS i. p. or diluent only. TNF-α was determined in plasma 2 h after challenge (A). One representative experiment of three is shown. n.d.: not detectable. IRF-7^−/−^ and TLR9^−/−^ mice were infected i. p. with 2×10^9^ Ad5 GFP particles and injected 16 h later with 1 µg LPS i. p. TNF-α was determined in plasma 2 h later (B). IFN-αβ signaling is required for Ad infection augmented LPS lethality. Wt and IFNαβR^−/−^ mice were infected with 1×10^10^ Ad5 GFP particles i. p. or left uninfected. 16 h later mice were injected with 3.5 µg LPS/gr. bw. The number of dead/total animals are shown at the indicated time after LPS challenge (C). Ad infection increases mCD14 expression on splenic macrophages in an IFN-αβ signaling dependent manner. Wt and IFN-αβR^−/−^ mice were infected with 1×10^10^ Ad5 GFP particles i. p. or left uninfected. Expression of mCD14 was measured on the surface of F4/80^+^ splenic macrophages by FACS 16 h after infection (D). Ad infection increases acetylation of histone H4 in wt macrophages at the TNF promoter. Wt and IFNαβR^−/−^ BMMs were mock-infected or infected with 5400 Ad5 GFP particles/cell and levels of acetylation of histone H4 were measured at the TNF-α promoter with ChIP assays (E). Representative experiments of two are shown.

Induction of type I IFNs was shown to be critical for some innate immune responses to Ads [10].We therefore investigated whether a characteristic consequence of infection with Ad, the induction of LPS hypersensitivity [72], might be mediated by type I IFNs. For this purpose, we infected wt and IFNαβR^−/−^ mice with Ads, challenged them 16 h later with LPS and measured the TNF-α response. Non-infected LPS-treated mice served as a control. Unlike the infected wt mice, the Ad3-infected IFNαβR^−/−^ mice did not exhibit enhanced responses to LPS (Fig. 11A). Interestingly, LPS hypersensitivity developed also in mice injected with very small amounts of Ad. Such amounts were capable of the elicitation of IFN-β mRNA in the spleen of infected animals, but incapable of inducing detectable circulating IFN-αβ (Fig. S14A and B). Similar results were obtained in Ad5-GFP-infected mice (not shown). Furthermore, in IRF-7^−/−^ mice that exhibit a severe impairment of the IFN-αβ response to Ad a severe impairment of LPS sensitization by Ad5-GFP was also observed. (Fig. 11B). In contrast, sensitization to LPS developed normally in Ad5-GFP-infected TLR9^−/−^ mice (Fig. 11B), which is in agreement with our finding that TLR9 is not critical for the induction of IFN-αβ by Ad. Furthermore, only Ad5-GFP-infected wt, but not IFNαβR^−/−^ mice exhibited enhanced susceptibility to LPS shock (Fig. 11C). Notably, also Ad vector-infected or IFN-α treated human monocyte-derived mDCs exhibited enhanced sensitivity to LPS and overproduced TNF-α upon LPS challenge (Fig. S14C). On the whole, our results indicate that IFN-αβ is an essential mediator of the LPS hypersensitivity induced by adenovirus infection.

An increased expression of the LPS receptor complex on target cells, may contribute to the enhanced reactivity to LPS [73],[74]. We therefore investigated whether macrophages of Ad infected mice overexpress the receptor components mCD14 and TLR4/MD-2. We found that splenic macrophages from Ad infected mice overexpress mCD14 in an IFN-αβ dependent manner (Fig. 11D) but the expression of TLR4/MD-2 was only minimally affected (Fig. S14D). Furthermore, we found increased acetylation levels of histone H4 at the TNF-α promoter in Ad-infected wt, but not in IFNαβR-deficient macrophages (Fig. 11E). This suggests that Ad-induced IFN-αβ increases LPS-induced TNF-α production, at least in part by epigenetic changes at the TNF-α promoter.

## Discussion

In the present study we investigated the IFN-αβ response of mice infected with human Ads. We showed that the response is characterized by high levels of IFN-α and IFN-β, which are produced simultaneously and almost exclusively by splenic mDCs. Furthermore, the response is entirely independent of viral replication, TLR-, MyD88-, TRIF- and IRF-3-signaling, but dependent on viral escape from the endosomes, activation of the SAPK/JNK pathway and IRF-7, and subsequent IFN-αβ feedback signaling. These data suggest an IFN-αβ induction pathway that is different from the extracytoplasmic and cytoplasmic pathways so far described. Finally, we show that IFN-αβ is a key mediator of the hypersensitivity to bacterial lipopolysaccharide, which develops, in Ad-infected mice.

### Adenoviruses, DCs and other IFN-αβ producing cell types

As shown previously and in this study, a wide spectrum of cells including pDCs, mDCs and macrophages [10],[45],[46],[63] produce IFN-αβ in response to Ad *in vitro*. The present finding that *in vivo*, in Ad-infected mice, splenic mDCs *are* the major source of IFN-αβ is surprising, since in mice infected with different viruses (MCMV, HSV, VSV, MHV, influenza, vaccinia and Sendai viruses) [27],[57],[58],[59],[75],[76],[77],[78] pDCs activated by the TLRs constitute the major contributors to the systemic levels of type I IFNs. In the present study the expression levels of IFN-αβ mRNAs in organs and cells from Ad-infected mice suggested a dominant role for splenic mDCs in the IFN-αβ response. Furthermore, the IFN response to Ad was practically absent in mice depleted of CD11c^high^ MHC II^+^ myeloid DCs. Also, there was a striking similarity between the IFN-αβ subtypes induced by Ads in the spleen of infected mice and those induced in mDC cultures *in vitro*. It should be emphasized that in mice infected with VSV, in which splenic pDCs are the main IFN producers, the spectrum of *in vivo* induced IFN-α subtypes was markedly different [76]. Finally, the finding that the response of Ad-infected mice was completely independent of TLR signaling and strongly dependent on IFN-αβ feedback provide further arguments against a major role for pDCs. In a number of viral infection models, IFN-α and -β production by pDCs was mediated by TLR7/9 [13],[19],[23] and was at least partially independent of a positive IFN-αβ feedback [76],[77],[79],[80].

In variance to our data, pDCs and various types of non-pDCs [10],[42] were suggested to be responsible for the type I IFN responses to adenoviral vectors in mice. In [10] the loss of TLR9 signaling resulted in a reduction of the IFN-α response in mice infected with the recombinant species C Ad-lacZ [10]. This finding suggested a significant contribution of TLR9, and therefore of pDCs, to the Ad-induced IFN-αβ response *in vivo*. The ratio of pDCs to mDCs (approximately 1∶1) in the spleen of animals used in the above study was quite different from that we and others [50] have found (approximately 1∶10). This may explain the conflicting results on the role of TLR9 *in vivo*, between this previous study and ours. In another study [42] the induction of IFN-αβ was studied in mice with an artificially enlarged pool of DCs (due to prior pretreatment with sFLT-3L), 24 h after recombinant Ad administration. In our study naïve mice were used and 24 h after Ad infection the levels of IFN-αβ were already below the detection limit. Of note also, the bone marrow stromal antigen 2 (BST2) (that is recognized by the PDCA-1 antibody used for isolation of pDCs in [42]), was shown to be up-regulated on numerous cell types following stimulation that triggers an IFN response [81]. Thus, the use of the PDCA-1 antibody for the isolation of pDCs seems to be more reliable in the case of uninfected mice [81].

Recently, an absolute IRF-3 dependency of the *in vitro* IFN-αβ response of bone marrow derived macrophages to Ad has been reported [63]. In our study IRF-3 deficiency had no significant effect on the levels of IFN-αβ induced in Ad-infected mice. In addition, in Ad-infected macrophages, the relatively low level of IFN-α4 mRNA and its negative regulation by the autocrine feedback was different from our *in vivo* findings (strong induction and positive regulation by feedback). This is in agreement with the concept that *in vivo* macrophages make no significant contribution to the IFN-αβ response to Ad. A likely explanation is that the induction of IFN-αβ in macrophages requires the high numbers of virions used *in vitro*, and that such multiplicities were never reached *in vivo*. Interestingly however, in contrast to IFN-αβ induction, the induction of IL-6 proceeded in the spleen of infected mice in both DC and non-DC populations. In accordance, the depletion of IFN-αβ-producing mDCs in mice prior to infection lowered, but did not entirely prevent the IL-6 response. It is possible that the pathways leading to the induction of IFN-αβ and IL-6 by Ad are different, at least in different cell types. Alternatively, *in vivo*, part of the IL-6 formed is induced indirectly *via* secondary mechanisms. In this context it is interesting that blockade of the p38 MAPK in mDCs *in vitro* had no effect on Ad-induced IFN-αβ production, but partially inhibited the production of IL-6. Our data showing that IFNαβR^−/−^ mice exhibit strongly impaired IL-6 responses to Ad is in agreement with a previous report [10] and shows that IFN-αβ is a positive regulator of IL-6 production. Moreover, our data indicate that this effect could be explained at least in part by the positive regulatory effect of IFN-αβ on Ad-induced IL-6 transcription and is achieved by the alteration of the chromatin structure at the IL-6 promoter.

### Requirements for IFN-α/β induction by adenoviruses and its relation to known induction pathways

The experiments carried out in this study in MyD88-, TRIF-, Unc93B and various TLR-deficient mice excluded a participation of TLRs in the IFN-αβ responses to Ads, including the recombinant Ad5-GFP. The only exception was the TLR9- and MyD88-dependent IFN-αβ response elicited by Ad2Ts1, a mutant virus deficient in endosomal escape [5]. However, compared to all other Ads used in this study, Ad2Ts1 induced very low levels of IFN-αβ, with faster kinetics and only when used in very high amounts. This is consistent with the finding that the mechanisms of type I IFN induction by Ad2Ts1 and the other adenoviruses are not the same. We suggest that the fast escape of Ads from the endosome circumvents activation of endosomal TLRs. The requirement for a longer endosomal retention time in TLR9-dependent IFN-αβ production has been recently demonstrated [82]. On the whole, our experiments indicate that Ad endosomal escape is required for the induction of IFN-αβ *in vivo* and suggest a cytosolic pathway. The same requirement was ascertained in the present study for the IL-6 response.

Sensors of nucleic acids are powerful initiators of the TLR-independent cytosolic IFN-αβ induction [12],[14],[15]. We excluded in this study a major involvement of the cytoplasmic RNA sensors RIG-I and MDA-5 in the induction of IFN-αβ by Ads. Likewise, our study does not support a major participation of the cytosolic DNA sensor DAI/Zbp1 in this induction either. However, our study did not formally exclude the existence of all potentially redundant recognition pathways. So far reported, the pathway(s) of IFN-αβ induction activated by cytosolic DNA is (are) strictly dependent on IRF-3 and minimally on IRF-7 [34],[35],[36],[37],[39]. IRF-3 was also reported to be essential for the adenoviral DNA-dependent induction of IFN-αβ in BMMs *in vitro* [63]. In agreement, in this study we show that transfection of BMDCs with naked adenoviral DNA or with whole virions of the endosomal escape deficient Ad2Ts1 results in a strictly IRF-3 dependent IFN-αβ response. Evidently however, the IFN-αβ response of Ad-infected BMDCs and mice is independent of IRF-3. These findings do not exclude a requirement for dsDNA recognition in the induction of IFN-αβ, but suggest a different induction mechanism and show the importance of cellular compartmentalization during normal Ad entry. A possible important factor involved in cytosolic Ad sensing might be the adenoviral cysteine protease L3/23, whose activation requires the presence of Ad DNA [83]. This assumption is supported by our finding that a small fraction of the protease-lacking Ad2Ts1 still reaches the cytosol (Fig. 10F, H), but is devoid of any IFN-αβ-inducing activity. This enzyme, apart from its involvement in maturation of viral proteins and endosomal escape, is essential for the stepwise disassembly of Ads in the cytosol and the release of viral DNA at the nuclear pore [5].

The absolute requirement of IRF-7 for type I IFN induction in Ad-infected mice shows that this transcription factor participates not only in the positive IFN-αβ feedback, but also in the initial IFN-αβ production and indeed plays a master role in the regulation of type I interferon response [29].

A further finding of the present study is the essential role for the MAPK SAPK/JNK in the IFN-αβ induction by Ads *in vitro* and *in vivo*, although the DNA-mediated cytosolic induction of IFN-αβ has been reported to be independent of MAPKs activation [14],[15],[36]. A cytosolic dsDNA-signaling pathway, mediated by the RNA helicase RIG-I and MAVS, and leading to the induction of IFN-β has been recently demonstrated in human hepatoma cells [84]. This induction pathway is absent in murine systems [22],[37],[84],[85] and is therefore unlikely to participate in the IFN-αβ response of Ad-infected mice.

On the whole, our findings do not support the participation of any known nucleic acid-mediated mechanisms in the elicitation of IFN-αβ responses to human Ads. In this context it is interesting that, as shown here, mouse embryonal fibroblasts do not produce IFN-αβ upon Ad infection, although they posses efficient cytosolic induction pathways for dsRNA or dsDNA [13],[36],[37], the latter shown also in this study. Rather, our data support the possibility that the IFN-αβ response to Ads occurs via a novel, not yet characterized cytosolic DNA or protein recognition pathway. Further studies are required to identify the viral components and the host receptors involved. We also emphasize that the known extra- and intra-cytosolic induction pathways may contribute to the IFN-αβ response in a host in which Ads replicate and free viral dsRNA and dsDNA are generated.

As mentioned above, the production of IFN-αβ in Ad-infected mice is strongly dependent on IFN-αβ feedback signaling. Inhibition of positive IFN-αβ feedback is a likely explanation for the negative regulation of the IFN-αβ response, observed, in mice infected with intact Ads (expressing early genes) in this study. Likely candidates for negative regulators of the IFN-αβ production are the E1A proteins. Previously, they were shown to inhibit Stat1 signal transduction [86], which plays a role in positive feedback signaling by means of the IFN-αβ receptor.

### Adenoviruses, toxic effects and therapeutic applications

Because the adenoviral vectors used in this study correspond to some of those used in gene therapy trials, the present findings in mice may have important implications for Ad gene therapy applications. The adverse effects observed in therapeutic trials, such as systemic inflammatory response and toxicity [6],[7],[9] can be at least partly explained by the enhanced susceptibility of the Ad-infected host to microbial components, such as LPS [72] and lipopeptides (unpublished results) from incoming secondary pathogens or from the patient's own flora. Our *in vitro* finding of a strongly enhanced TNF-α response to LPS in Ad5-GFP-infected human DCs suggests that enhanced susceptibility to LPS may develop also in patients treated with adenoviral vectors. As shown here, this hypersensitivity is mediated by viral-induced IFN-αβ, which is in accordance with the role of IFN-αβ as a key mediator of sensitization to LPS [87],[88]. The increased mCD14 expression on LPS target cells and epigenetic changes on promoters of relevant genes (both shown here), may at least in part explain the role of IFN-αβ in the development of Ad induced LPS hypersensitivity.

Type I interferon induction was recently found in recombinant Ad-treated human cells, especially in pDCs [46],[89] and in monocyte-derived DCs in the present study. Moreover, this response was observed also in patients administered with recombinant adenoviral vectors [42],[90]. Since as shown here, IFN-α pre-treatment is capable of increasing susceptibility to LPS of human DCs in vitro, we assume that Ad-induced IFN-αβ can induce LPS hypersensitivity in humans *in vivo*. Undesirable complications mediated by IFN-αβ can occur not only during gene therapy, but also in immunocompromised patients where Ads are major pathogens [1]. Our finding that blocking SAPK/JNK signaling inhibits the IFN-αβ response to Ads, is of potential interest for prevention or treatment of the direct and indirect adverse effects of IFN-αβ in Ad gene therapy.

## Materials and Methods

### Mouse strains

Wt C57BL/6, C57BL/10 and 129Sv mice, as well as all knockout mice were bred under SPF conditions at the MPI. Breeding pairs of IRF-3^−/−^ and IRF-7^−/−^ mice were kindly provided by T. Taniguchi and K. Honda, (Department of Immunology, Graduate School of Medicine, University of Tokyo, Tokyo), of the TRIF and Unc 93B^−/−^ mice by B. Beutler (Scripps Research Institute, La Jolla) and of MyD88^−/−^ mice by M. Kopf (TH Zürich) and R. Landmann (Department Forschung, Kantonspital Basel). MDA-5^−/−^ femurs were provided by M. Colonna (Washington University School of Medicine, St. Louis). IRF-3- [30], IRF-7- [29], Myd88- [91], TRIF- [92], TLR-9- [93], Unc 93B [94] MDA-5 [25] deficient mice and CD11c-diphtheria toxin receptor (DTR)/GFP transgenic mice [54] were on a C57BL/6 background, TLR2/4-deficient mice [95] on C57BL/10 background and IFNαβR-deficient mice [96] on 129Sv and for the lethality experiments on C57BL/6 background. When the strain of the mouse was not indicated C57BL/6 mice were used. Mice of both sexes, 8-12 weeks of age were used for the experiments. All of the experimental procedures were in accordance with institutional, state and federal guidelines on animal welfare.

### Viruses

Human Ads of species B (Ad serotype 3) and C (Ad R700, an Ad serotype 5 derivative, Ad serotype 2, Ad2Ts1 and Ad5-GFP an early gene expression defective Ad were grown, purified and stored as previously described [5],[72]. The ratio of infectious/total viral particles was determined on susceptible cells and was typically 1∶20–50. If not otherwise stated, Ad2Ts1 used for the experiments was grown at non-permissive temperature 38.5°C (resulting in the absence of incorporation of the adenoviral protease L3/23 into viral capsids). Empty particles were identified by their light density in CsCl density gradients and the absence of viral DNA and were purified simultaneously with mature virions. UV inactivation of Ad was done as described previously [5],[72] using the minimal essential dose preventing viral gene expression and replication in susceptible cells. Heat inactivation was done at 56 °C for 60 min [44]. All virus preparations were LPS-free (less than 1 pg LPS/10^11^ viral particles) as determined by the Limulus amebocyte lysate test (Pyroquant Diagnostic GMBH, Mörfelden, Germany).

### Cell culture, inhibitors, plasmids and transfection

MEFs, BMMs and GM-CSF induced BMDCs were generated as described [95],[97]. The purity of BMMs was higher than 98%, of BMDCs approximately 80%. BM derived pDCs were generated in the presence of Flt3L and CD11c^+^ CD11b^−^ B220^+^ CD62L^+^ pDCs were MoFlo sorted (purity higher than 95%) as described [55]. MEFs were grown in α-MEM (Invitrogen). Immature, monocyte derived human DCs were obtained by incubating adherent monocytes with GM-CSF as described [98] Human mDCs were infected with the indicated amounts of Ads in growth medium containing 2% of donor serum. Mouse L-929 cells were grown in DMEM with 10% FCS. MAPK inhibitors UO126 (MEK1/2, Cell Signaling), SB203580 (p38, Sigma) and SP600125 (SAPK/JNK Calbiochem) were used at 15 µM *in vitro* and SP600125 at 20 mg/kg *in vivo*. Bafilomycin-A1 (Sigma) was used at 100 nM. The plasmids pmaxGFP (Amaxa) and RIG-IC [64],[99],[100] were purified with the Endo-Free Plasmid kit (Qiagen) and PEG purification. RV leader RNA was generated by in vitro transcription (MEGA shortscript Kit; Ambion) from a synthetic DNA template: 5′ACATTTTTGCTTTGCAATTGACAATGTCTGTTTTTTCTTTGATCTGGTTGTTAAGCGTTATAGTGAGTCGTATTACGCG-3′ annealed with 5′-AATTCGCGTAATACGACTCACTATA-3′. RNA was purified using mini Quick Spin RNA Columns (Roche). siRNA mediated knockdown of DAI/Zbp1 was done using DAI/Zbp1 targeting siRNAs (On-Target Plus Smartpool reagent, Dharmacon). For the induction of type I IFNs MEFs and BMDCs were transfected with nucleic acids complexed with Lipofectamine 2000 (Invitrogen) in 96-well plates according to the instructions of the manufacturer. L-929 cells were cotransfected with siRNAs and pmaxGFP using Lipofectamine 2000 according to the suggestions of the supplier. Subsequently GFP positive cells were purified by FACS sorting for further analysis. BMDCs were co-transfected with siRNAs or RIG-IC together with pmaxGFP using the nucleoporator apparatus and the mouse dendritic cell nucleofector kit (Amaxa) according to the suggestions of the supplier and gene expression was analyzed in FACS-sorted GFP positive cells.

### Spleen cell fractionation, FACS sorting and depletion of DC subsets

Splenocytes of 3–5 spleens were separated into CD11c^+^ and CD11c^−^ cells by magnetic adsorption cell sorting (MACS; Miltenyi Biotec). Both fractions were further purified by FACS sorting using anti CD11c-biotin and Streptavidin-PE-Cy5 (BD PharMingen). For the analysis and isolation of splenocytes and CD11c^+^ subsets, anti-CD11c-biotin, anti-mouse CD11b-Alexa Fluor 647, Gr-1 FITC, B220 PE, F4/80 PE, anti-mouse CD14- Alexa Fluor 647 and anti-mouse TLR4/MD2-Alexa Fluor 647 antibodies and Streptavidin-PE-Cy5 (BD PharMingen) and anti-Siglec H-biotin from Hycult Biotechnology were used. 4–20×10^4^ cells in the different fractions were sorted using MoFlo (Dako Cytomation, Glostrup,) to a typical purity of 95%–97%. To deplete mDCs, CD11c-DTR/GFP mice were injected i.p. with DT (4 ng/g body weight; Sigma-Aldrich) as described. pDCs were depleted from naïve mice with 500 µg i.p. injected rat anti–mPDCA-1 mAb (Miltenyi Biotec) 24 h prior to Ad infection.

### Detection of secreted cytokines and intracellular proteins

Murine IFN-αβ activity was measured using an L-929 cell line (provided by B. Beutler and Z. Jiang, Scripps Research institute, La Jolla) as described [18] Human IFN-αβ bioactivity was measured using the HL116 cells from G. Uzé as described [101]. The contribution of IFN-α or IFN-β to the total IFN-αβ activity was determined by pre-incubating plasma for 1 h with excess amounts of neutralizing anti–IFN-β antibody (Yamasa Corporation, Japan) or control antibody. Murine TNF-α was measured by a bioassay as described [95]. IL-6 was detected with an ELISA from Pharmingen BD. Human TNF-α was detected with an ELISA from R&D Biosystems. JNK/SAPK MAPKs were detected on immunoblots with antibodies detecting all or phosphorylated isophorms of the proteins (Cell Signaling). Zbp1/DAI and β-actin were detected on immunoblots with antibodies from Santa Cruz Biotechnology.

### Conventional RT-PCR, real-time RT-PCR, analysis of IFN-áâ subtypes and Chromatin Immunoprecipitation

Total RNA was isolated from organs and cells with guanidinium-thiocyanate-phenol-chloroform extraction or with TRI reagent (Sigma). To exclude DNA contamination, RNA samples were treated with RNase free DNase I (Fermentas). cDNA was prepared using Expand reverse transcriptase (Roche) and oligo-dT. Conventional RT-PCRs were performed with primers as follows. β-actin: GTC CAC ACC CGC CAC CAG TTC G and GGA ATA CAG CCC GGG GAG CAT CGT C, IFN-β: CCT TTG CAC CCT CCA GTA ATA G and GAC GGA GAA GAT GCA GAA GAG T, IFN-α: ATG GCT AGR CTC TGT GCT TTC CT and AGG GCT CTC CAG AYT TCT GCT CTG, Ad2 E1A: GTT ATT ACC GAA ATG GCC GCC AGT CT and CTT CGG GGG CCG TCA CGT CTA AAT CAT AC.

Real-time PCR quantification of RNA expression was done using the LightCycler II system (Roche) and the Quantitect SYBR Green PCR Kit (Qiagen) according to the instructions of the manufacturers. Arbitrary units of relative expression were generated by dividing the value obtained for IFN-α, -β or IL-6 by the value of β-actin and multiplying the result by 1000. The detection limits for the IFN-α and IFN-β PCRs were 150 and 30 copies/reaction, respectively. The following primers were used for real-time RT-PCR: β-actin: TGG AAT CCT GTG GCA TCC ATG AAA and TAA AAC GCA GCT CAG TAA CAG TCC G, IFN-β: CCT TTG CAC CCT CCA GTA ATA G and GAC GGA GAA GAT GCA GAAGAG T, IFN-α: TCT GAT GCA GGT GGG and AGG GCT CTC CAG ACT TCT GCT CTG, IL-6: GTG ACA ACC ACG GCC TTC CCT AC and TGC AAG TGC ATC ATC GTT GTT CAT, Zbp1/DAI: GAC GAC AGC CAA AGA AGT GA and GAG CTA TGT CTT GGC CTT CC. The IFN-α primers are consensus sequences from previous publications [102],[103], detecting all IFN-α mRNAs. To determine the levels of different IFN-αβ subtypes the HT7900 quantitative PCR system (Applied Biosystems) was used. cDNAs were measured in duplicates or triplicates using the following gene-specific assays (TaqMan Gene Expression Assays, Applied Biosystems): IFN-alpha2 (Mm00833961_s1), IFN-alpha4 (Mm00833969_s1), IFN-alpha5 (Mm00833976_s1), IFN-alpha6 (Mm02524285_g1), IFN-alpha9 (Mm00833983_s1), IFN-alpha11 (Mm01257312_s1), IFN-alpha12 (Mm00616656_s1), IFN-alpha13 (Mm00781548_s1), IFN-alpha14 (Mm01703465_s1),IFN-beta (Mm00439546_s1). The gene for mouse hypoxanthine guanine phosphoribosyl transferase-1 (HPRT-1, Mm00446968_m1) was used to calibrate the mRNA levels. Quantitative analysis was performed using the SDS 2.1 software (Applied Biosystems). mRNA levels were calculated by the following formula: relative expression = 2∧(−(Ct(Target)-Ct(Endogenous control))*f, with f = 10 000 as an arbitrary factor.

Chromatin Immunoprecipitation (ChIP) assays were performed as described [104]. Briefly, cells were cross-linked with 1% formaldehyde and sonicated nuclear extracts were mock-immunoprecipitated or immunoprecipitated with anti-tetraacetylated histone H4 (Upstate). Recovered DNA aliquots from these samples and from input extracts were amplified with real-time PCR using the LightCycler II system (Roche) and Quantitect SYBR Green PCR Kit (Qiagen). Enrichments at specific chromatin loci are shown as the amount of immunoprecipitated DNA in the percent of total input chromatin. The following primers were used: IL-6 promoter: TGG GGA TGT CTG TAG CTC ATT and CAT AGC GGT TTC TGG AAT TGA, TNF promoter: GGG CAG CCC CAG AGG GAA TGA ACT C and TAT GGC AGA GGC TCC GTG GAA AAC TCA CT, Topoisomerase 3β promoter: AGT CCG AGA ACA GCC TGG GT and AGT TGT GCT GCC CAC AGA GG, λ5 promoter: TCC CCA TTG CCA GAT AGA GAC ACA and TGG GCC CAA CAG ATT AAC ACA GAG.

### Transmission electron microscopy

BMDCs were cold synchronized with saturating amounts of Ad2 and Ad2-ts1 (60 µg/ml, 0.25 ml per 4×10^4^ cells on a 12 mm glass coverslip) for 1 h, washed and incubated at 37°C for the indicated times. The samples were fixed with 2.5% glutaraldehyde in 0.1 M ice-cold Na-Cacodylate buffer (pH 7.2) containing 0.5 mg/ml ruthenium red for 1 h, washed with 0.1 M Na-Cacodylate buffer (pH 7.2), post-fixed with 2% OsO4 in the same buffer containing 0.5 mg/ml ruthenium red for 1 h at room temperature, and embedded in Epon as described [105]. Virus particles at the plasma membrane, endosomes and the cytosol were determined, and results expressed as means of analyzed cells (n) with standard errors of the mean.

### Data analysis and statistics

Data was analyzed using Prism GraphPad 4.0 software. Data in all figures are presented as mean, error bars show SEM. Statistical analysis was performed with the unpaired t-test (*: P<0.05; **: P<0.01; ***: P<0.005).

### List of accession numbers/ID numbers for proteins mentioned in the text

DEFINITION interferon-beta.
   ACCESSION AAA72488
DEFINITION interleukin 6 [Mus musculus].
   ACCESSION NP_112445
DEFINITION tumor necrosis factor [Mus musculus].
   ACCESSION NP_038721
DEFINITION CD14 [Mus musculus].
   ACCESSION CAA32166
DEFINITION toll-like receptor 9 [Mus musculus].
   ACCESSION NP_112455
DEFINITION toll-like receptor 4 [Mus musculus].
   ACCESSION NP_067272
DEFINITION toll-like receptor 2 [Mus musculus].
   ACCESSION NP_036035
DEFINITION MyD88 [Mus musculus].
   ACCESSION AAC53013
DEFINITION TRIF [Mus musculus].
   ACCESSION BAA76376
DEFINITION interferon induced with helicase C domain 1 (MDA-5) [Mus musculus].
   ACCESSION NP_082111
DEFINITION DEAD/H box polypeptide RIG-I [Mus musculus].
   ACCESSION NP_766277 XP_990501
DEFINITION Z-DNA binding protein 1 [Mus musculus].
   ACCESSION NP_067369

## Supporting Information

Figure S1 IFN-αβ induction in response to Ads. Induction kinetics of IFN-αβ in the plasma of B6 mice (4–6/group) after the indicated time-points of i. p infection with 3.6×10^10^ viral particles of intact Ad R700. 6 h and 8 h values were analyzed for statistical significance using the unaired t-test. (A). Control of UV inactivation of Ads. B6 mice were infected intraperitoneally with Ad3 or UV inactivated Ad3 and 1.2×10^10^ particles/mouse and the expression of Ad E1A mRNA was measured in the spleen and liver 16 h after infection. BMDCs were infected with native or inactivated Ad *in vitro* with (5400 particles/cell) and analyzed for E1A mRNA expression 8 h after infection (B). BMDCs were infected in vitro with Ad5 GFP and UV inactivated Ad5 GFP (5400 particles/cell). The expression of GFP was controlled by FACS analysis 16 h after infection (C).
(311 KB TIF)

Figure S2 IFN-αβ and IL-6 induction in response to Ads. Kinetics of the IFN-αβ response to UV inactivated Ad R700 (A) or Ad5 GFP (B) in B6 mice. Groups of B6 mice (4–6/group) were infected i.p. with 3.6×10^10^ viral particles or of UV inactivated Ad R700 (A) or Ad5 GFP (B) and plasma for IFN measurement was collected at the indicated time-points. IFN-αβ and IL-6 responses to graded doses of Ad R700. B6 mice (4–6/group) were infected with 4×10^9^ (1), 1.2×10^10^ (2), 3.6×10^10^ (3) and 7.2×10^10^ (4) of native (empty bars) or UV-inactivated (filled bars) Ad R700 viral particles/mouse, i.p. Mice were bled 6 hours after infection and the levels of IFN-αβ (C) and IL-6 (D) in plasma were determined. Representative experiments of two are shown. Effiect of heat inactivation on Ad entry. BMDCs from B6 mice were infected with native or heat inactivated Ad3 (5400 particles/cell) and the presence of internalized Ad DNA was detected with PCR 16 h later (E) in nuclear extracts as described [66].
(203 KB TIF)

Figure S3 Induction of IFN-αβ in pDCs and in human DCs. IFN-αβ response of *in vitro* generated mouse mDC and pDC to Ad3. BM derived GM-CSF- induced mDCs and FLt3L-induced pDCs generated from B6 mice were mock-infected or infected with 600, 1800 and 5400 viral particles of Ad3/cell. Cell-free supernatants for IFN-αβ measurement were collected 16 h after infection (A). Absence of IFN-αβ induction in L-929 cells by Ad. L-929 cells were infected with 1800 and 5400 Ad2 particles/cell or left uninfected. IFN-αβ was measured in cell-free supernatants 16 h after infection (B, left). The expression of E1A and β-actin mRNAs was measured 16 h after Ad2 infection by RT-PCR (B, right). IFN-αβ response of human primary DCs to Ad. Human monocyte derived DC were mock-infected or infected with of Ad5 GFP or Ad3 (16 200 particles/cell). IFN-αβ was measured in cell-free supernatants 24 h after infection. (C)
(181 KB TIF)

Figure S4 Purification and analysis of lymphocyte subsets in the spleen of Ad infected mice. Groups of B6 mice (4–6/group) were infected i.p. with 3.6×10^10^ viral particles of Ad3 and spleens for analysis were removed 8 h after infection. Pooled splenocytes were analyzed with the respective antibodies by FACS. Gates used for the analysis and purification of splenocytes and DC subsets are shown for representative samples. FACS sorting of DC subsets (A–D) To isolate pDCs, CD11c^+^ MACS enriched cells were subdivided as CD11c^int^, CD11b^−^ cells (A) and then Gr-1^+^, B220^+^ (B) cells. mDCs were isolated from CD11c^+^ cells (A) as CD11b^+^, Gr-1^−^ cells (C). From CD11c^+^, CD11b^−^ cells (C) a F4/80^+^ subpopulation was obtained (D).
(252 KB TIF)

Figure S5 Analysis of DC depletion after diphteria toxin (DT) treatment in the spleen of CD11c-DTR and B6 mice. CD11c-DTR and B6 mice (4 animals/group) were injected with DT i. p or left untreated. 24 h later the animals were infected with 1.2×10^10^ viral particles of Ad3 and DC populations in splenocytes from individual animals were analyzed with flow cytometry using the indicated antibodies. The percentage of the respective population in a representative animal is given in the plot panels (A–B).
(704 KB TIF)

Figure S6 Analysis of pDC depletion after anti mPDCA-1 antibody treatment. Mice were untreated (−αPDCA-1; 4 animals/group) or injected with 500 µg of anti-mPDCA-1 antibody (+αPDCA-1; 4 animals/group) injected with Ad3 and the pDC (A) and mDC (B) populations were analyzed in splenocytes of individual mice with flow cytometry. Representative data for one mouse/group are shown.
(524 KB TIF)

Figure S7 Intracellular TLR signaling is not required for Ad5-GFP induction of IFN-αβ. Wt and TLR9 −/− or Unc93B −/− mice (4 animals/group) were infected with 1×10^10^ Ad5 GFP particles i. p. and IFN-αβ was determined in plasma 6 h after infection (A). Cytokine response of various TLR deficient mice to TLR ligands. Wt, TLR9, MyD88, TRIF and TLR2/4 deficient mice were challenged with 10 nmol CpG ODN 1668 or with 1 µg of LPS i.p. as indicated. IFN-αβ was measured in plasma 2 h after stimulation (B, left). Wt and TLR2/4 deficient mice were injected with 40 µg of lipopeptide i. p. (Pam3CysK4) and plasma TNF-α levels were measured 2 h after challenge (B, right).
(162 KB TIF)

Figure S8 Role of IFN-αβ feedback signaling in Ad-infected BMDCs. BMDCs from wt and IFNαβR −/− mice were mock-infected or infected with 600, 1800 and 5400 Ad3 particles/cell. IFN-αβ was measured in cell-free supernatants 16 h after infection. One representative experiments of three is shown.
(75 KB TIF)

Figure S9 IRF-7 but not IRF-3 is required for Ad5GFP induced IFN-αβ production *in vivo*. Wt, IRF-3 and IRF-7 deficient mice were injected with 1×10^10^ particles of Ad5 GFP i. p. and plasma IFN-αβ levels were measured 6 h after infection.
(98 KB TIF)

Figure S10 Knock-down of DAI/Zbp1 in L-929 cells. L-929 were transfected with pmaxGFP alone (4 µg DNA/1.6×10^6^ cells) or together with DAI/Zbp1 targeting siRNAs (4 µg DNA and 200 pmol siRNA/1.6×10^6^ cells) with Lipofectamine 2000. Forty-two hours later the cells were stimulated with B-DNA: poly(dA-dT)·poly(dT-dA) (2 µg/ml) complexed with Lipofectamine 2000 for 6 hrs or left unstimulated and GFP^+^ cells were purified with FACS-sorting. Knockdown of DAI/Zbp1 mRNA (A) and protein (B) was measured with real-time RT-PCR and with immunoblotting, respectively. Expression of IFN-β mRNA was measured with real-time RT-PCR (C).
(179 KB TIF)

Figure S11 Adenovirus induced IL-6 production is dependent on JNK signaling. BMDC cultures from B6 mice were pretreated with the indicated MAPK inhibitors or with diluent for 15 min and then infected with 1800 and 5400 viral particles of Ad3/cell or were mock infected. IL-6 levels were measured from cell-free supernatants taken 6 h after infection. A representative experiment of three is shown.
(108 KB TIF)

Figure S12 The role of viral endosomal escape in adenovirus triggered cytokine responses. Empty Ad particles do not induce type I IFN *in vivo*. B6 mice (4/group) were infected with 1.2×10^10^ viral particles of mature Ad3 and with an equivalent dose of empty Ad3 particles. Plasma samples were taken 8 hours after infection and the levels of IFN-αβ were measured (A). Comparison of the induction of IL-6 of mice infected with wild-type Ad2 or the endosomal escape deficient Ad2Ts1 virus. B6 Mice (4/group) were infected with 3.6×10^10^ Ad2 or Ad2Ts1 grown at 38.5°C (Ts1), i. p. IL-6 was measured 4 and 8 h after infection in plasma samples. (B) Comparison of the cytokine responses of BMDCs infected with Ad2 or Ad2Ts1 (Ts1). BMDCs from B6 mice were infected with 1800, 5400, and 16 200 particles of Ad2/cell or with 32 400 particles of Ts1/cell or were mock-infected. IFN-αβ (C) and IL6 (D) were measured in cell-free supernatants 16 h after infection. Comparison of Ad vector and Ad2Ts1 induced IFN-αβ production of human monocyte derived DCs. Cells were infected with 16 200 particles of Ad5 GFP or Ad2 Ts1/cell or were mock-infected. IFN-αβ was measured in cell-free supernatants 24 h after infection (E).
(175 KB TIF)

Figure S13 Bafilomycin A1 inhibits Ad3 triggered IFN-αβ and IL-6 production *in vitro*. BMDC cultures derived from C57BL/6 mice were pretreated with the 100 nM bafilomycin A1 (black bars) or with diluent (empty bars) for 15 min and then infected with 1800, 5400, and 16 200 viral particles of Ad3/cell or were mock infected. IFN-αβ (A) and IL-6 (B) levels were measured from the cell-free supernatants taken 6 h after infection. Representative experiments of three are shown.
(127 KB TIF)

Figure S14 Adenovirus triggered LPS hypersensitivity. Low doses of Ads can induce LPS hypersensitivity. B6 mice (3/group) were infected with 5×10^8^ (1) or 5×10^9^ (2) particles of Ad5 GFP or left uninfected. Expression of IFN-β in spleen and plasma IFN-αβ was measured using RT-PCR and bioassay, respectively (A). In a separate experiment mice were infected as described above and 16 h later challenged with 1 µg of LPS i. p. TNF-α levels were measured in plasma 2 h later. (B). Ad infection and IFN-α treatment augments LPS induced TNF-α production of human DCs. Human monocyte derived DCs were mock-treated, or pre-infected with Ad5 GFP or Ad3 (16 200 particles/cell) or pre-treated with 50 U/ml hIFN-α. 16 h later the cells were stimulated with 1 µg/ml LPS overnight. TNF-α was measured in cell-free supernatants (C). Ad infection increases minimally the expression of TLR4/MD2 on splenic macrophages. Wt and IFN-αβR −/− mice were infected with 1×10^10^ Ad5 GFP particles i. p. or left uninfected. Expression of TLR4/MD2 was measured on the surface of F4/80^+^ splenic macrophages by FACS 16 h after infection (D).
(335 KB TIF)
