# Supplementary figures and images for: Key Role of Splenic Myeloid DCs in the IFN-αβ Response to Adenoviruses In Vivo
Source: PLoS Pathog. 2008 Nov 14;4(11):e1000208. doi: 10.1371/journal.ppat.1000208 (PMC2576454; doi:10.1371/journal.ppat.1000208)

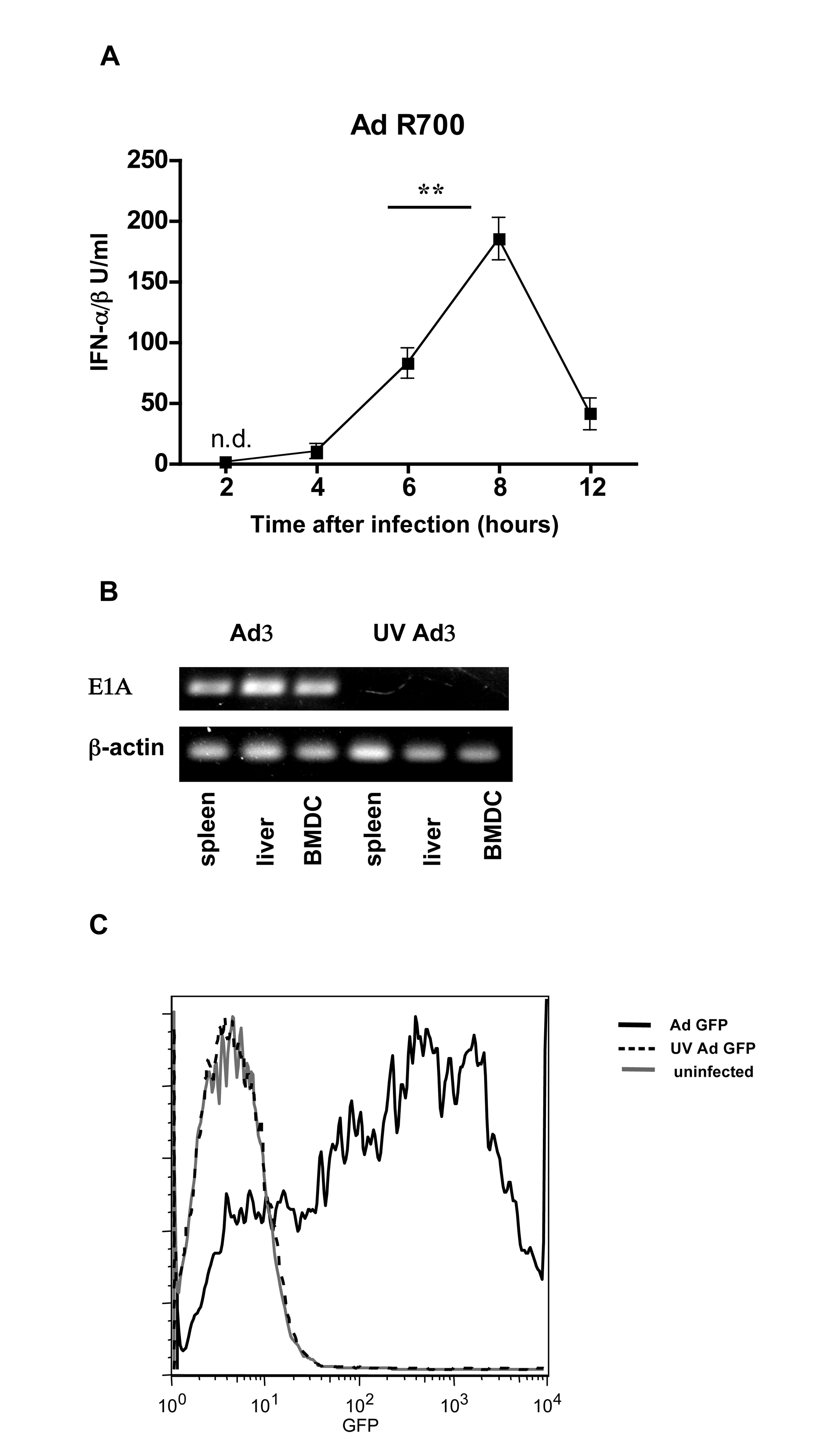

Supplement: Figure S1 — IFN-αβ induction in response to Ads. Induction kinetics of IFN-αβ in the plasma of B6 mice (4–6/group) after the indicated time-points of i. p infection with 3.6×10^10^ viral particles of intact Ad R700. 6 h and 8 h values were analyzed for statistical significance using the unaired t-test. (A). Control of UV inactivation of Ads. B6 mice were infected intraperitoneally with Ad3 or UV inactivated Ad3 and 1.2×10^10^ particles/mouse and the expression of Ad E1A mRNA was measured in the spleen and liver 16 h after infection. BMDCs were infected with native or inactivated Ad in vitro with (5400 particles/cell) and analyzed for E1A mRNA expression 8 h after infection (B). BMDCs were infected in vitro with Ad5 GFP and UV inactivated Ad5 GFP (5400 particles/cell). The expression of GFP was controlled by FACS analysis 16 h after infection (C). (311 KB TIF) [file ppat.1000208.s001.tif]

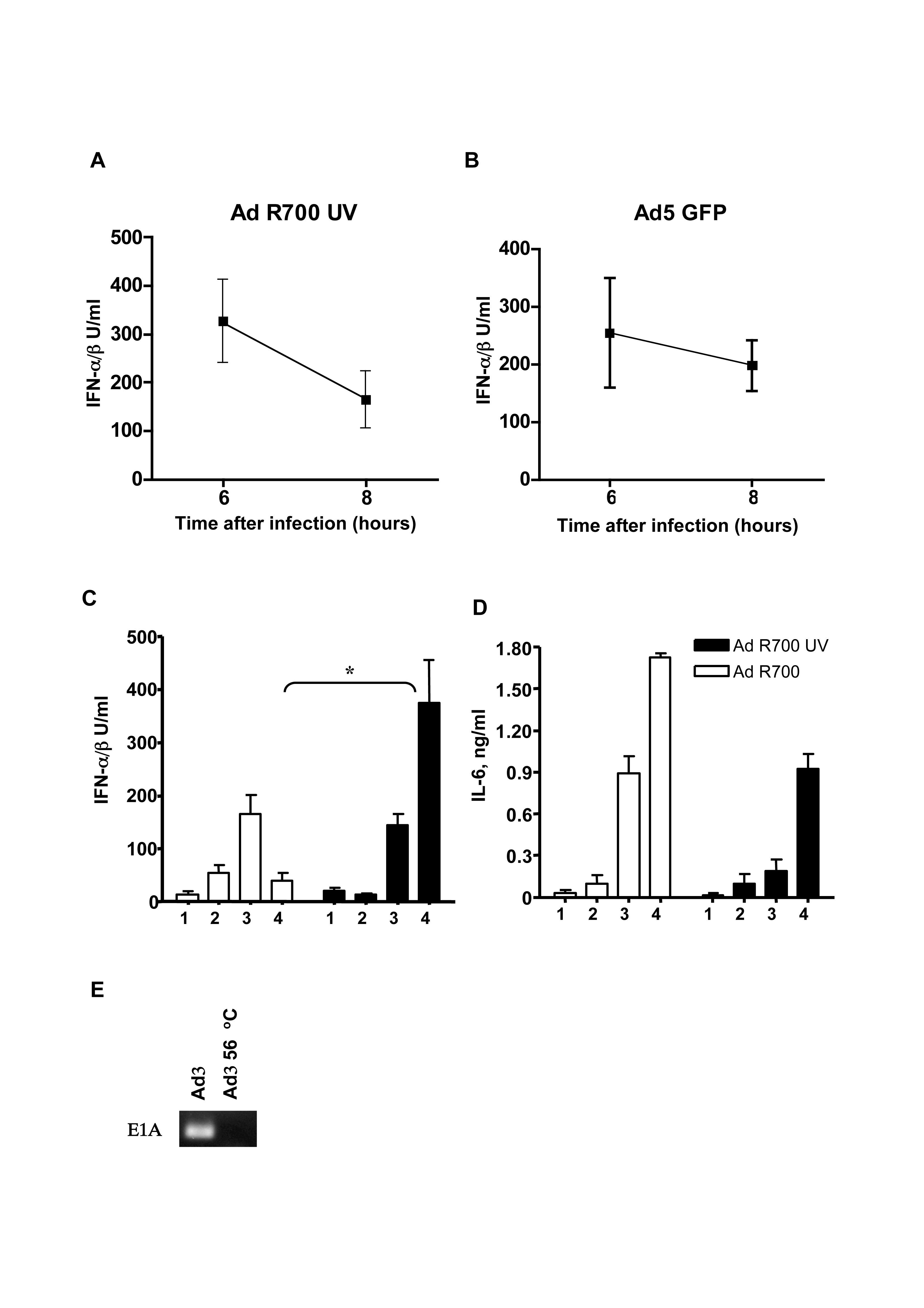

Supplement: Figure S2 — IFN-αβ and IL-6 induction in response to Ads. Kinetics of the IFN-αβ response to UV inactivated Ad R700 (A) or Ad5 GFP (B) in B6 mice. Groups of B6 mice (4–6/group) were infected i.p. with 3.6×10^10^ viral particles or of UV inactivated Ad R700 (A) or Ad5 GFP (B) and plasma for IFN measurement was collected at the indicated time-points. IFN-αβ and IL-6 responses to graded doses of Ad R700. B6 mice (4–6/group) were infected with 4×10^9^ (1), 1.2×10^10^ (2), 3.6×10^10^ (3) and 7.2×10^10^ (4) of native (empty bars) or UV-inactivated (filled bars) Ad R700 viral particles/mouse, i.p. Mice were bled 6 hours after infection and the levels of IFN-αβ (C) and IL-6 (D) in plasma were determined. Representative experiments of two are shown. Effiect of heat inactivation on Ad entry. BMDCs from B6 mice were infected with native or heat inactivated Ad3 (5400 particles/cell) and the presence of internalized Ad DNA was detected with PCR 16 h later (E) in nuclear extracts as described [66]. (203 KB TIF) [file ppat.1000208.s002.tif]

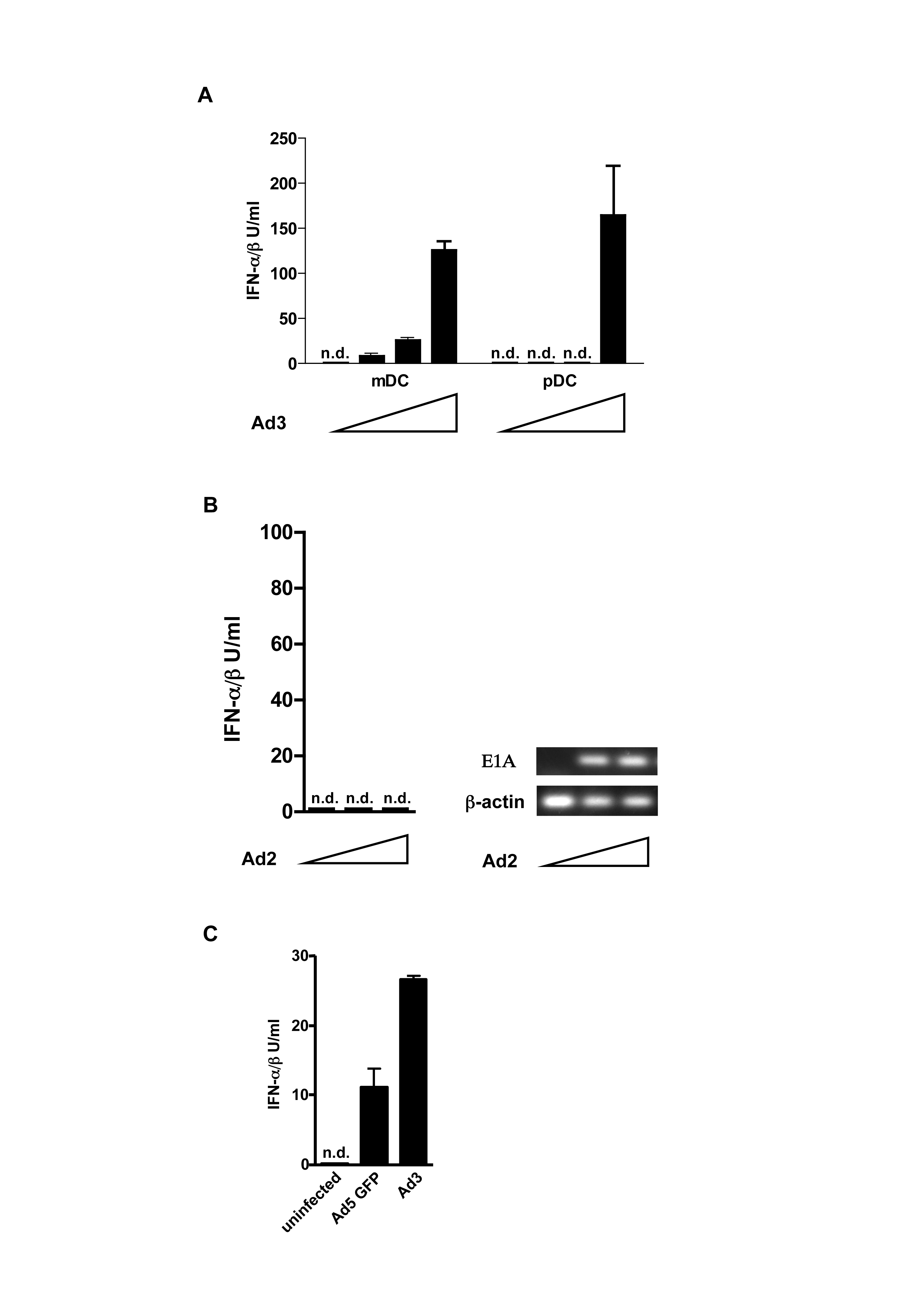

Supplement: Figure S3 — Induction of IFN-αβ in pDCs and in human DCs. IFN-αβ response of in vitro generated mouse mDC and pDC to Ad3. BM derived GM-CSF- induced mDCs and FLt3L-induced pDCs generated from B6 mice were mock-infected or infected with 600, 1800 and 5400 viral particles of Ad3/cell. Cell-free supernatants for IFN-αβ measurement were collected 16 h after infection (A). Absence of IFN-αβ induction in L-929 cells by Ad. L-929 cells were infected with 1800 and 5400 Ad2 particles/cell or left uninfected. IFN-αβ was measured in cell-free supernatants 16 h after infection (B, left). The expression of E1A and β-actin mRNAs was measured 16 h after Ad2 infection by RT-PCR (B, right). IFN-αβ response of human primary DCs to Ad. Human monocyte derived DC were mock-infected or infected with of Ad5 GFP or Ad3 (16 200 particles/cell). IFN-αβ was measured in cell-free supernatants 24 h after infection. (C) (181 KB TIF) [file ppat.1000208.s003.tif]

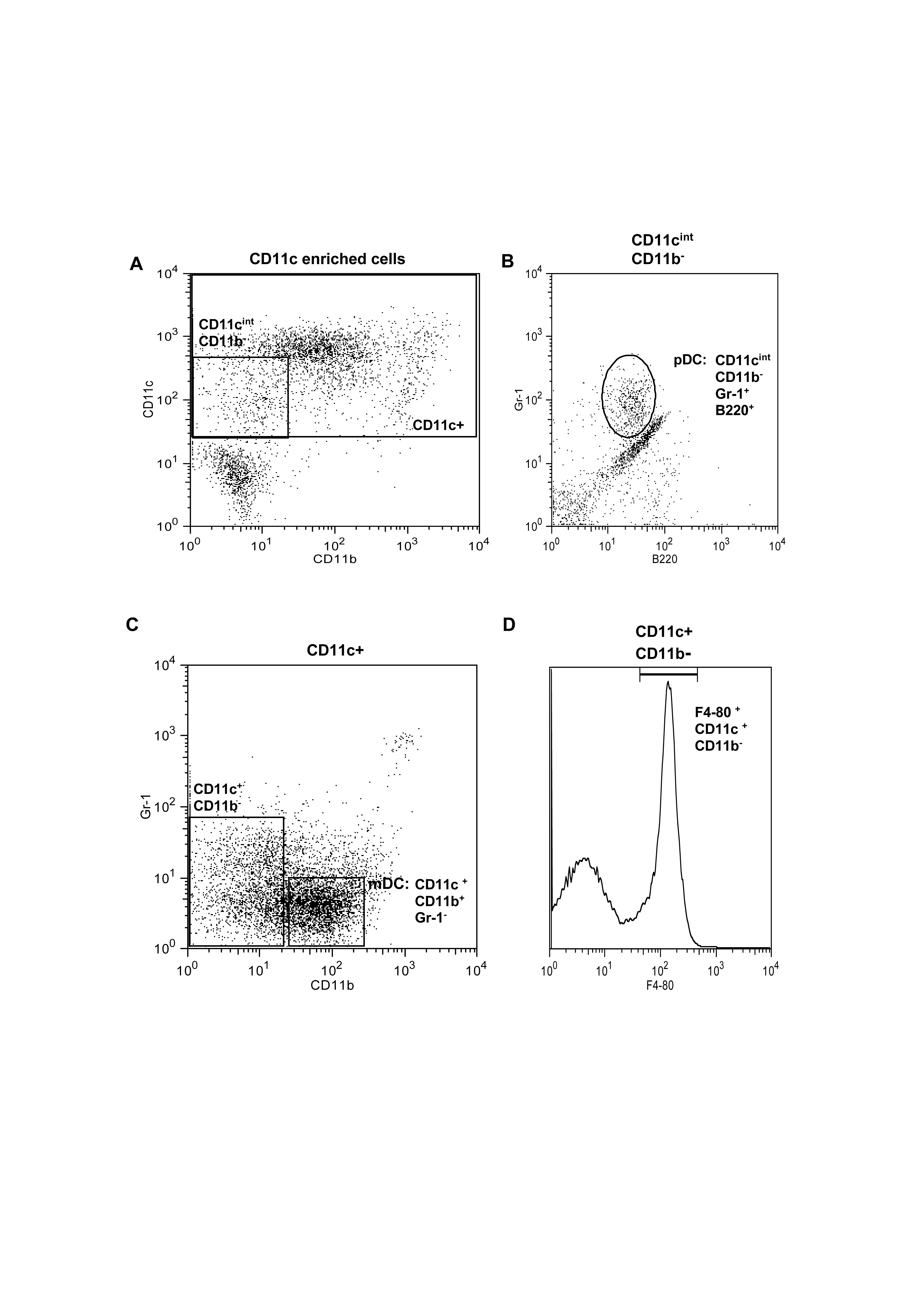

Supplement: Figure S4 — Purification and analysis of lymphocyte subsets in the spleen of Ad infected mice. Groups of B6 mice (4–6/group) were infected i.p. with 3.6×10^10^ viral particles of Ad3 and spleens for analysis were removed 8 h after infection. Pooled splenocytes were analyzed with the respective antibodies by FACS. Gates used for the analysis and purification of splenocytes and DC subsets are shown for representative samples. FACS sorting of DC subsets (A–D) To isolate pDCs, CD11c+ MACS enriched cells were subdivided as CD11cint, CD11b− cells (A) and then Gr-1+, B220+ (B) cells. mDCs were isolated from CD11c+ cells (A) as CD11b+, Gr-1− cells (C). From CD11c+, CD11b− cells (C) a F4/80+ subpopulation was obtained (D). (252 KB TIF) [file ppat.1000208.s004.tif]

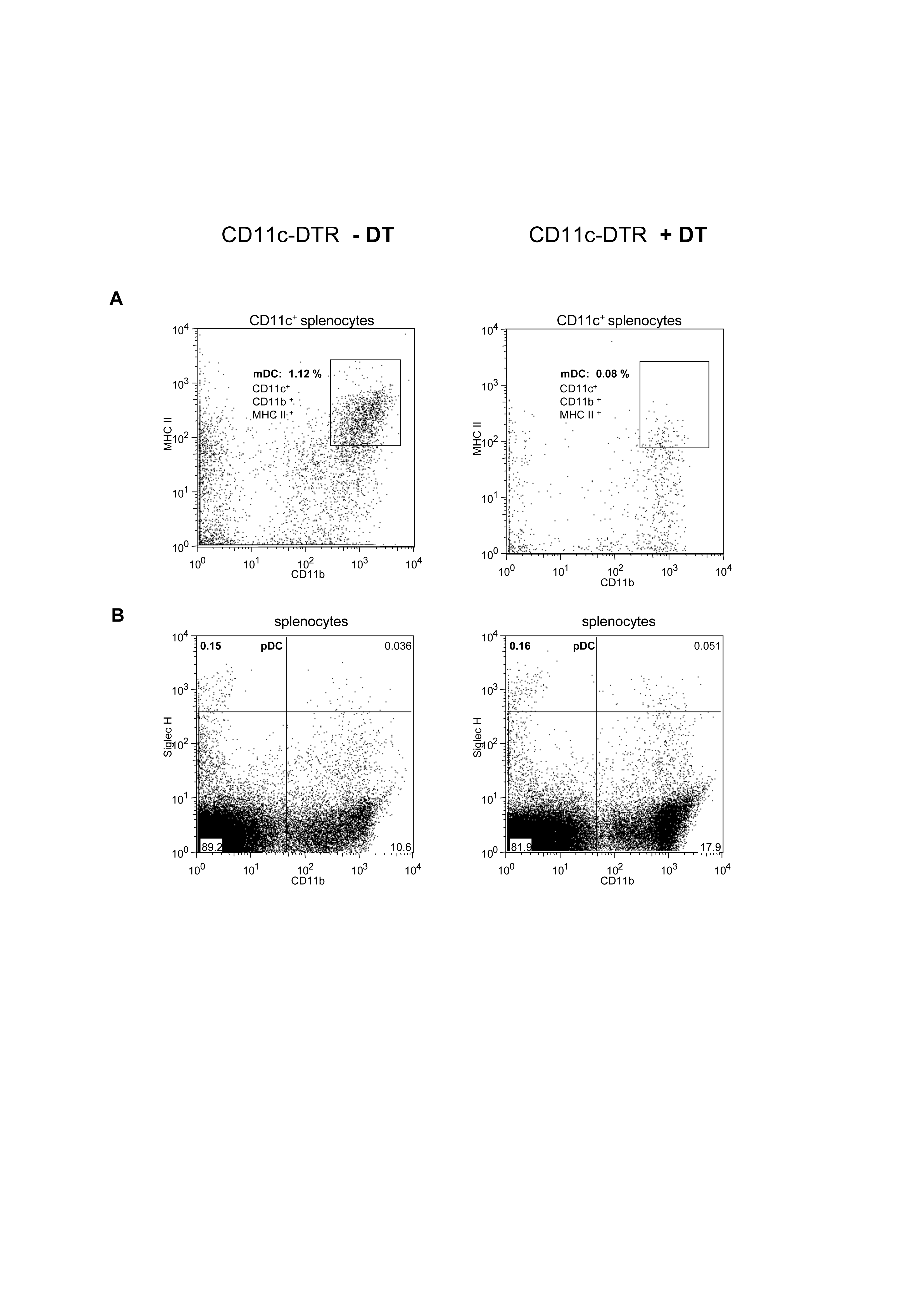

Supplement: Figure S5 — Analysis of DC depletion after diphteria toxin (DT) treatment in the spleen of CD11c-DTR and B6 mice. CD11c-DTR and B6 mice (4 animals/group) were injected with DT i. p or left untreated. 24 h later the animals were infected with 1.2×10^10^ viral particles of Ad3 and DC populations in splenocytes from individual animals were analyzed with flow cytometry using the indicated antibodies. The percentage of the respective population in a representative animal is given in the plot panels (A–B). (704 KB TIF) [file ppat.1000208.s005.tif]

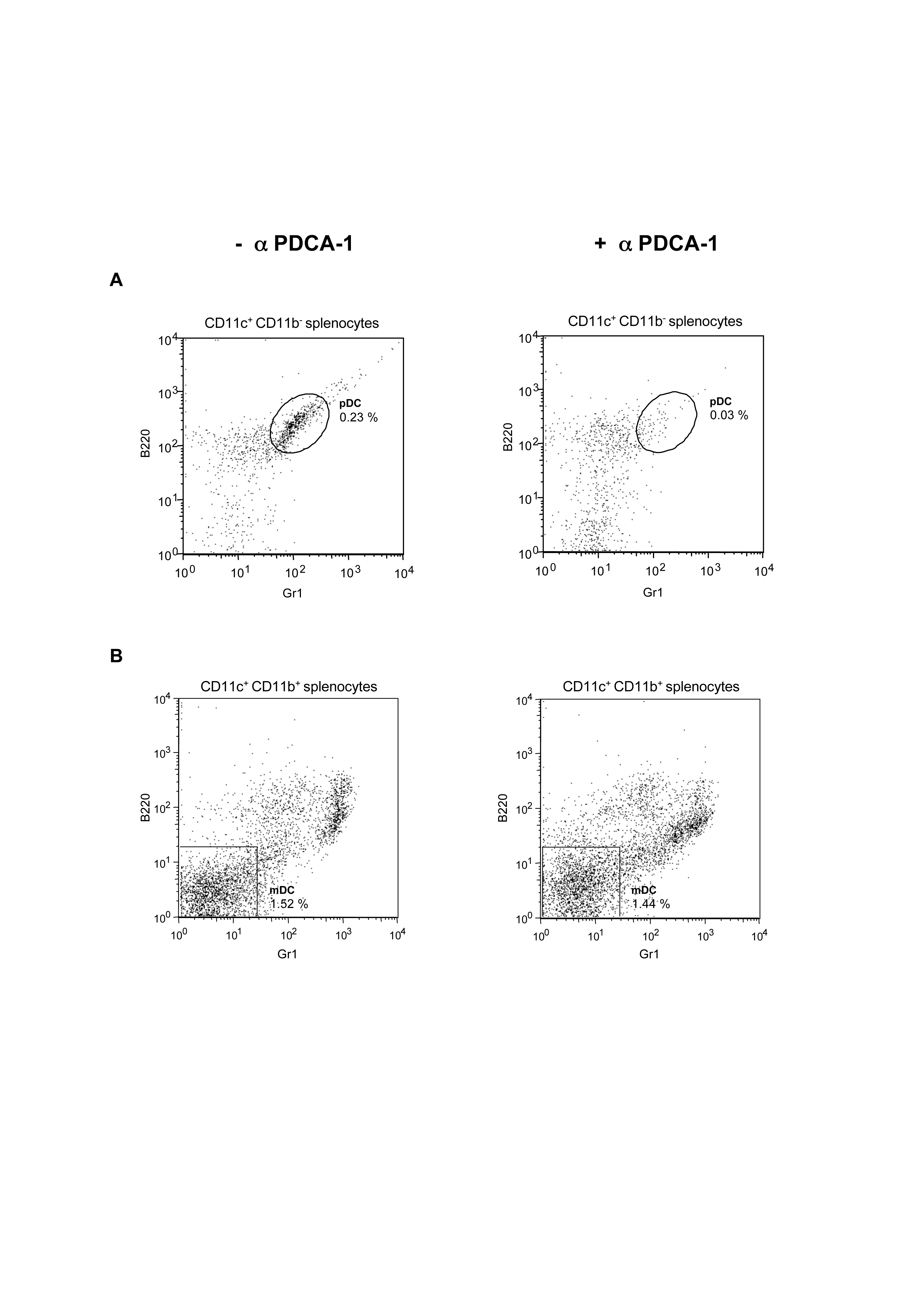

Supplement: Figure S6 — Analysis of pDC depletion after anti mPDCA-1 antibody treatment. Mice were untreated (−αPDCA-1; 4 animals/group) or injected with 500 µg of anti-mPDCA-1 antibody (+αPDCA-1; 4 animals/group) injected with Ad3 and the pDC (A) and mDC (B) populations were analyzed in splenocytes of individual mice with flow cytometry. Representative data for one mouse/group are shown. (524 KB TIF) [file ppat.1000208.s006.tif]

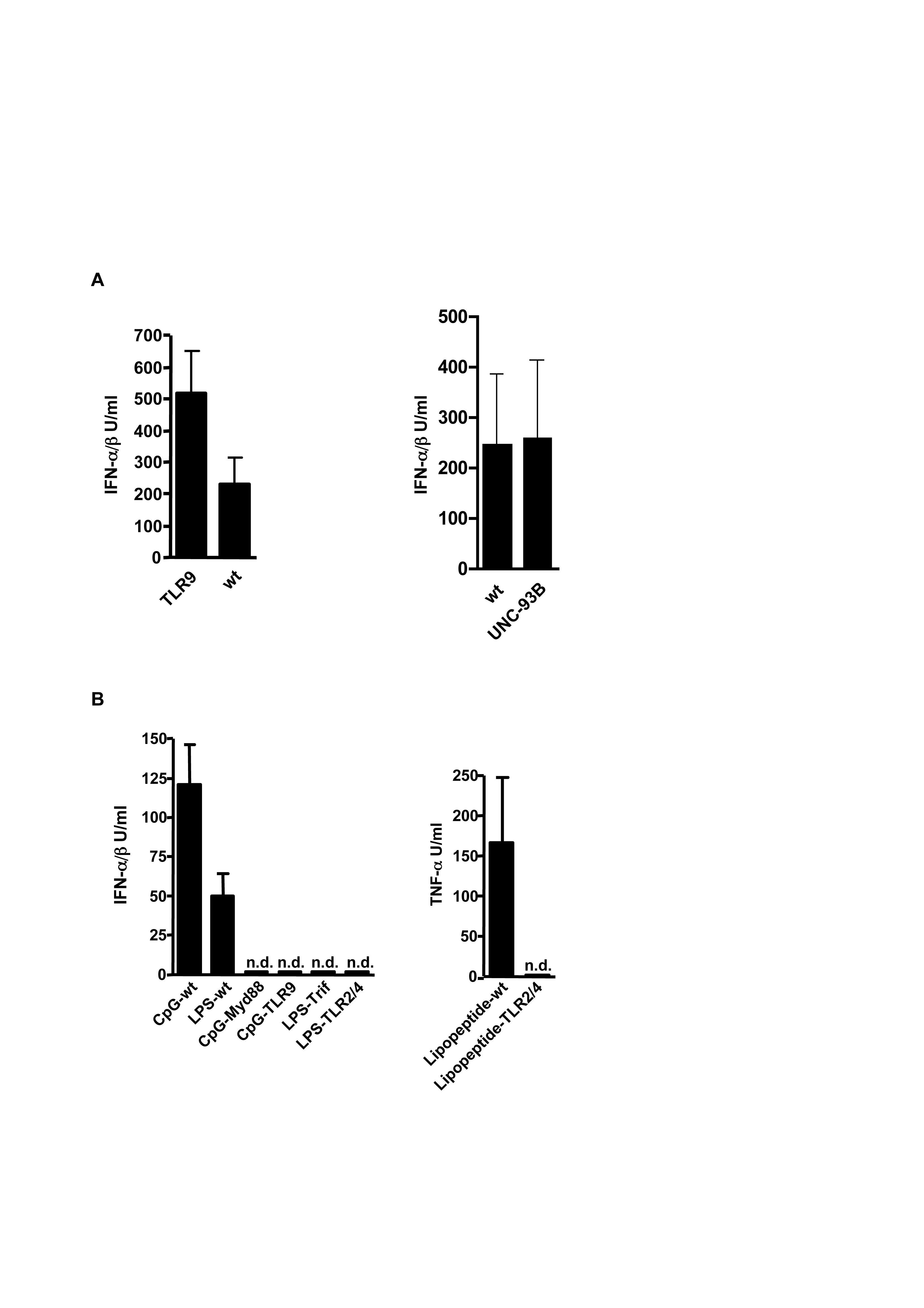

Supplement: Figure S7 — Intracellular TLR signaling is not required for Ad5-GFP induction of IFN-αβ. Wt and TLR9 −/− or Unc93B −/− mice (4 animals/group) were infected with 1×10^10^ Ad5 GFP particles i. p. and IFN-αβ was determined in plasma 6 h after infection (A). Cytokine response of various TLR deficient mice to TLR ligands. Wt, TLR9, MyD88, TRIF and TLR2/4 deficient mice were challenged with 10 nmol CpG ODN 1668 or with 1 µg of LPS i.p. as indicated. IFN-αβ was measured in plasma 2 h after stimulation (B, left). Wt and TLR2/4 deficient mice were injected with 40 µg of lipopeptide i. p. (Pam3CysK4) and plasma TNF-α levels were measured 2 h after challenge (B, right). (162 KB TIF) [file ppat.1000208.s007.tif]

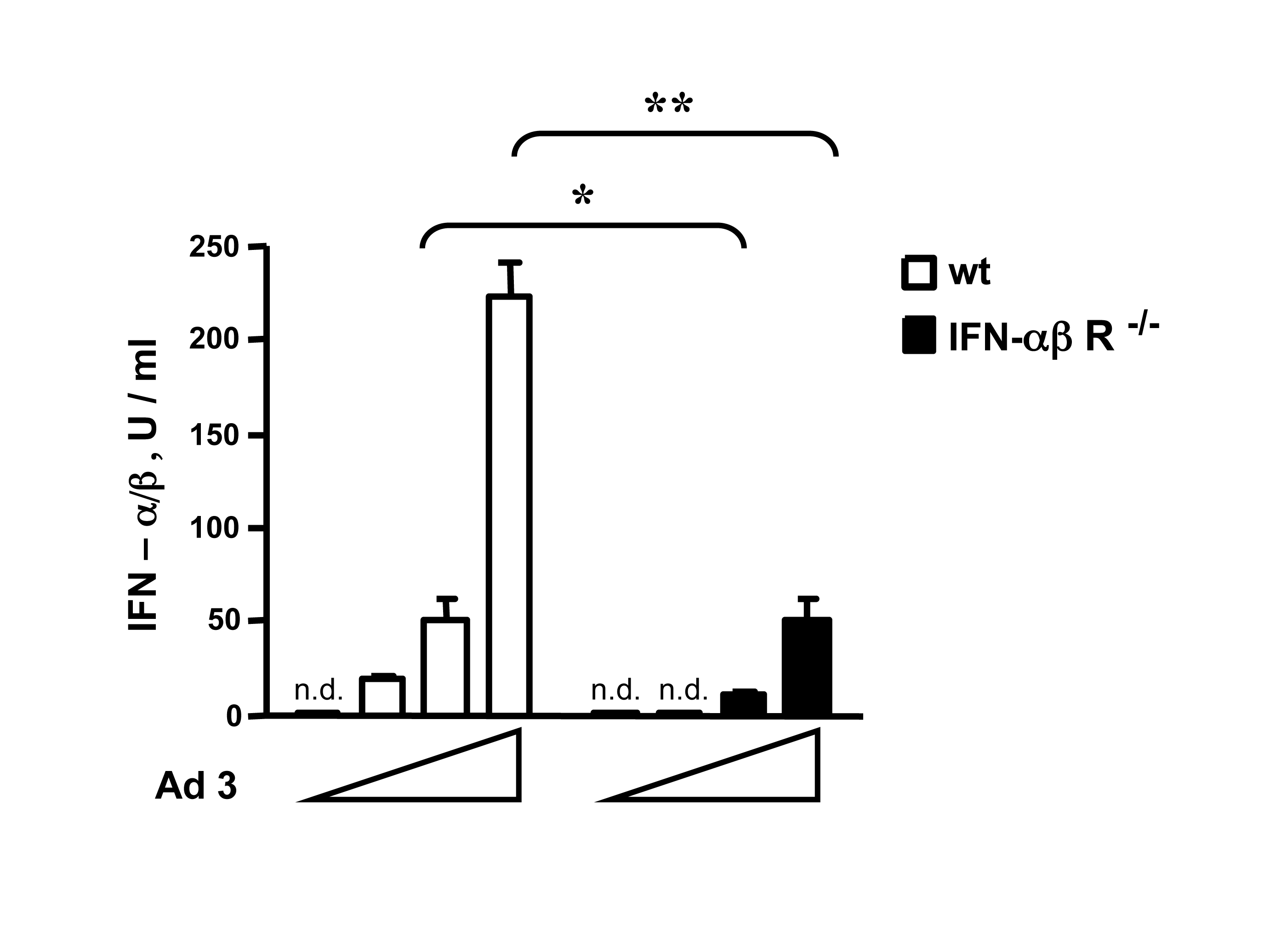

Supplement: Figure S8 — Role of IFN-αβ feedback signaling in Ad-infected BMDCs. BMDCs from wt and IFNαβR −/− mice were mock-infected or infected with 600, 1800 and 5400 Ad3 particles/cell. IFN-αβ was measured in cell-free supernatants 16 h after infection. One representative experiments of three is shown. (75 KB TIF) [file ppat.1000208.s008.tif]

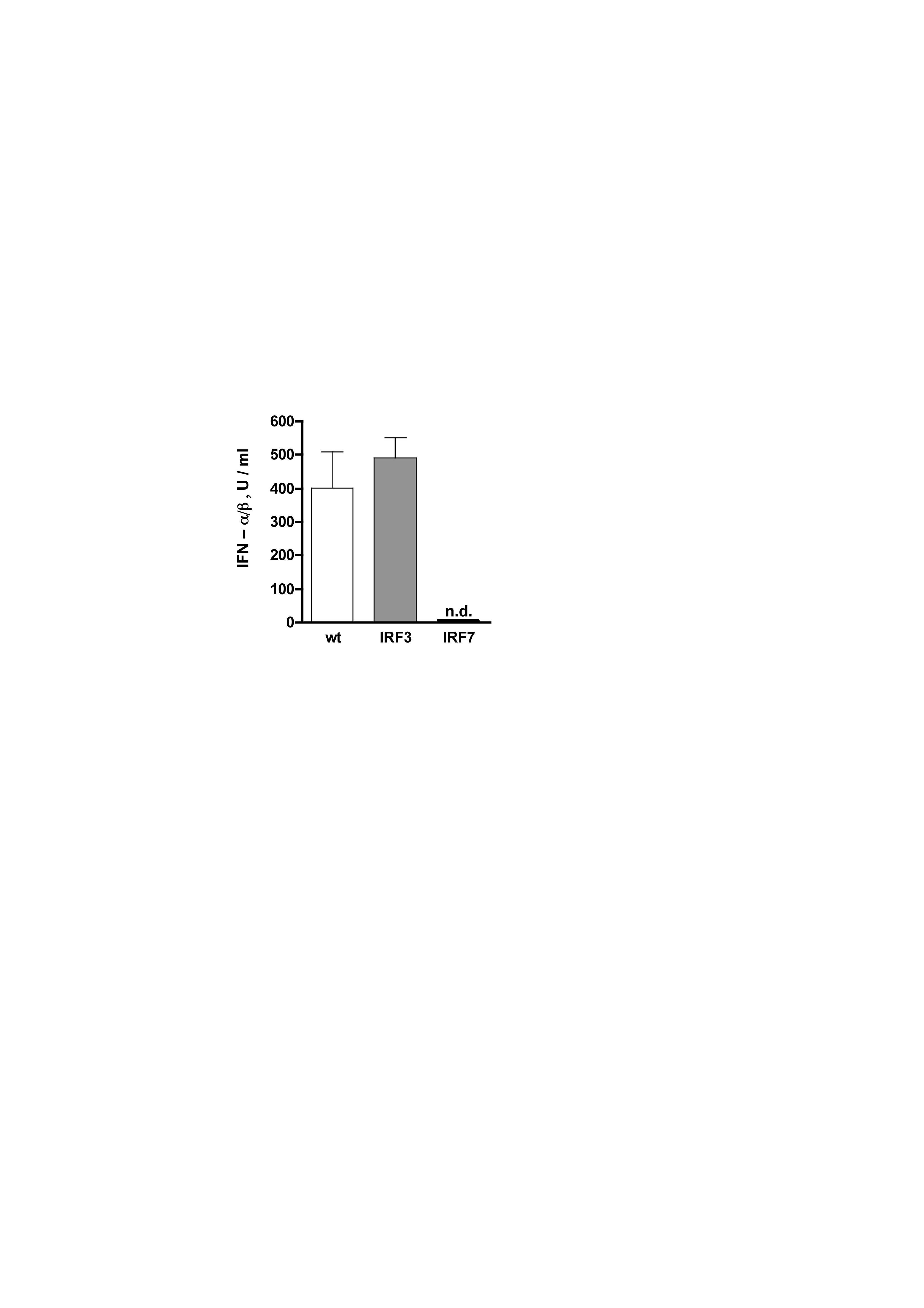

Supplement: Figure S9 — IRF-7 but not IRF-3 is required for Ad5GFP induced IFN-αβ production in vivo. Wt, IRF-3 and IRF-7 deficient mice were injected with 1×10^10^ particles of Ad5 GFP i. p. and plasma IFN-αβ levels were measured 6 h after infection. (98 KB TIF) [file ppat.1000208.s009.tif]

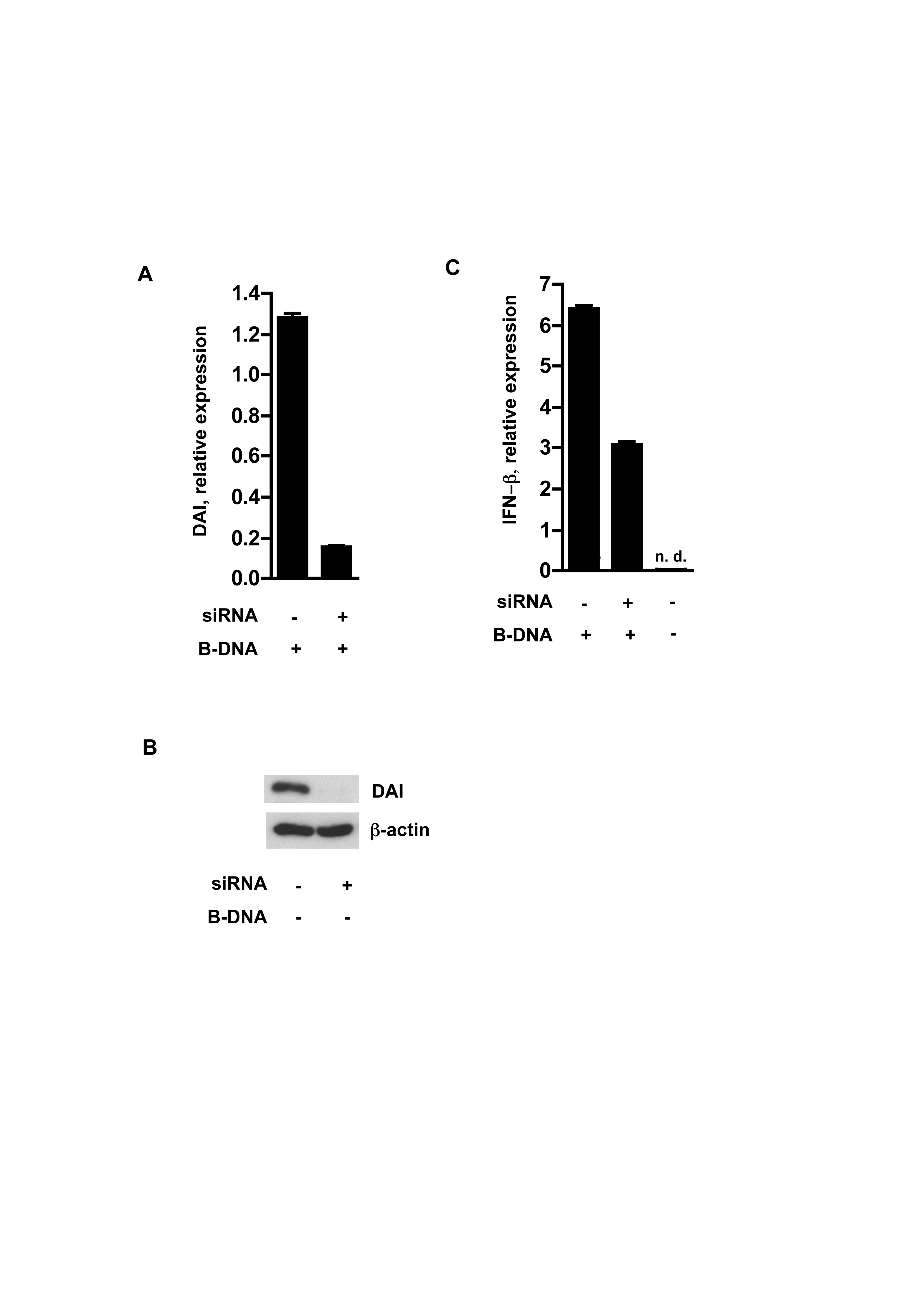

Supplement: Figure S10 — Knock-down of DAI/Zbp1 in L-929 cells. L-929 were transfected with pmaxGFP alone (4 µg DNA/1.6×106 cells) or together with DAI/Zbp1 targeting siRNAs (4 µg DNA and 200 pmol siRNA/1.6×106 cells) with Lipofectamine 2000. Forty-two hours later the cells were stimulated with B-DNA: poly(dA-dT)·poly(dT-dA) (2 µg/ml) complexed with Lipofectamine 2000 for 6 hrs or left unstimulated and GFP+ cells were purified with FACS-sorting. Knockdown of DAI/Zbp1 mRNA (A) and protein (B) was measured with real-time RT-PCR and with immunoblotting, respectively. Expression of IFN-β mRNA was measured with real-time RT-PCR (C). (179 KB TIF) [file ppat.1000208.s010.tif]

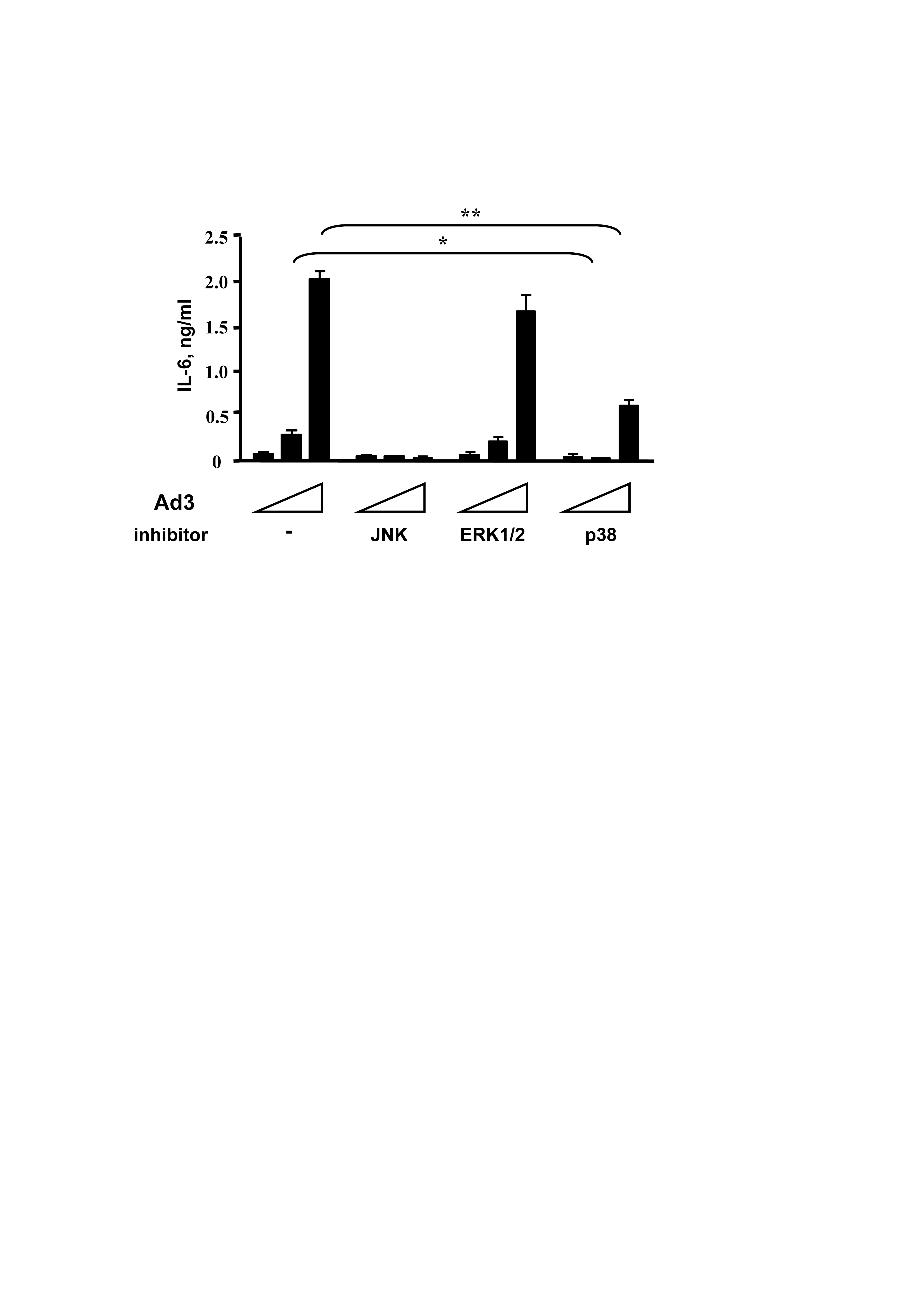

Supplement: Figure S11 — Adenovirus induced IL-6 production is dependent on JNK signaling. BMDC cultures from B6 mice were pretreated with the indicated MAPK inhibitors or with diluent for 15 min and then infected with 1800 and 5400 viral particles of Ad3/cell or were mock infected. IL-6 levels were measured from cell-free supernatants taken 6 h after infection. A representative experiment of three is shown. (108 KB TIF) [file ppat.1000208.s011.tif]

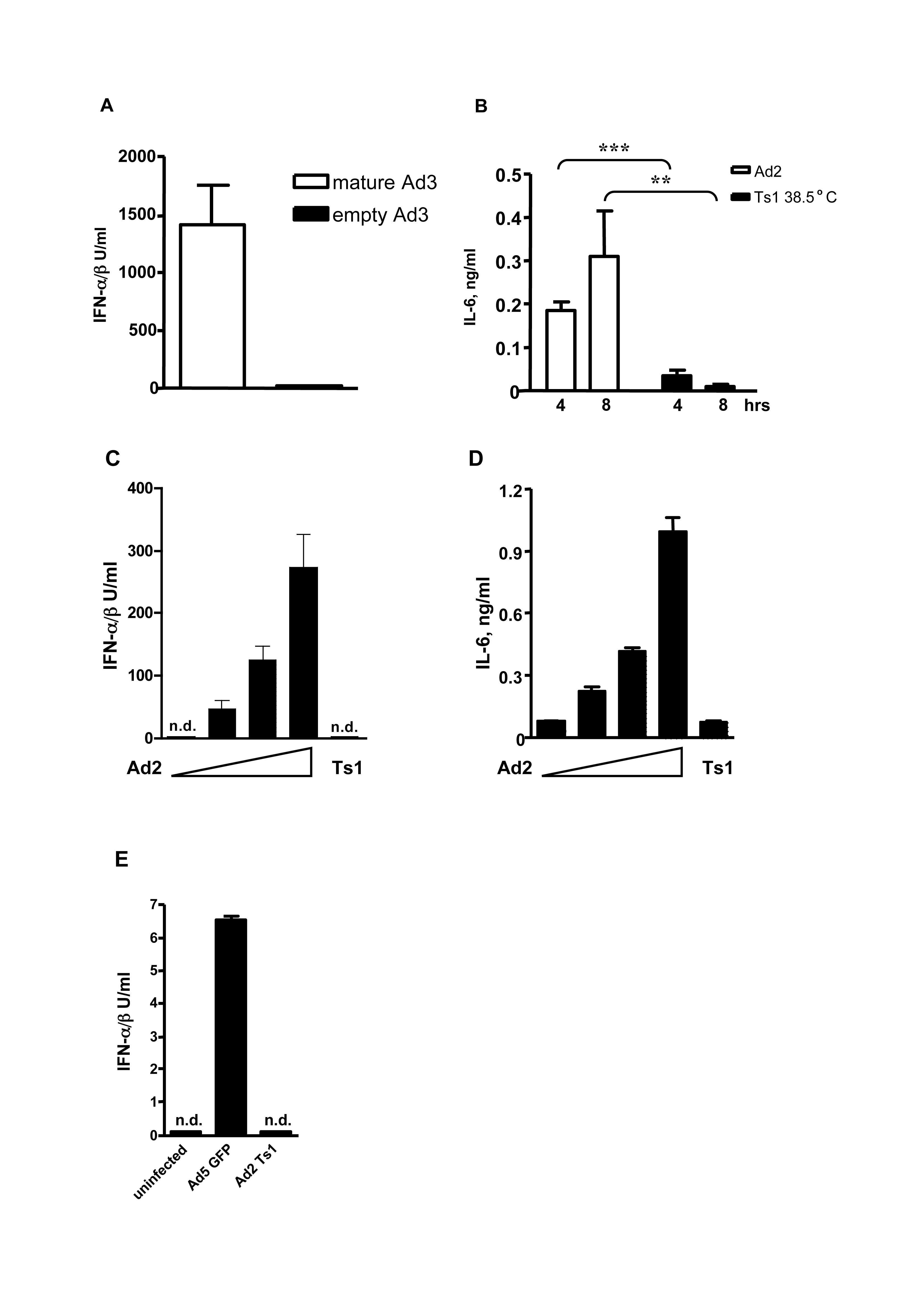

Supplement: Figure S12 — The role of viral endosomal escape in adenovirus triggered cytokine responses. Empty Ad particles do not induce type I IFN in vivo. B6 mice (4/group) were infected with 1.2×10^10^ viral particles of mature Ad3 and with an equivalent dose of empty Ad3 particles. Plasma samples were taken 8 hours after infection and the levels of IFN-αβ were measured (A). Comparison of the induction of IL-6 of mice infected with wild-type Ad2 or the endosomal escape deficient Ad2Ts1 virus. B6 Mice (4/group) were infected with 3.6×10^10^ Ad2 or Ad2Ts1 grown at 38.5°C (Ts1), i. p. IL-6 was measured 4 and 8 h after infection in plasma samples. (B) Comparison of the cytokine responses of BMDCs infected with Ad2 or Ad2Ts1 (Ts1). BMDCs from B6 mice were infected with 1800, 5400, and 16 200 particles of Ad2/cell or with 32 400 particles of Ts1/cell or were mock-infected. IFN-αβ (C) and IL6 (D) were measured in cell-free supernatants 16 h after infection. Comparison of Ad vector and Ad2Ts1 induced IFN-αβ production of human monocyte derived DCs. Cells were infected with 16 200 particles of Ad5 GFP or Ad2 Ts1/cell or were mock-infected. IFN-αβ was measured in cell-free supernatants 24 h after infection (E). (175 KB TIF) [file ppat.1000208.s012.tif]

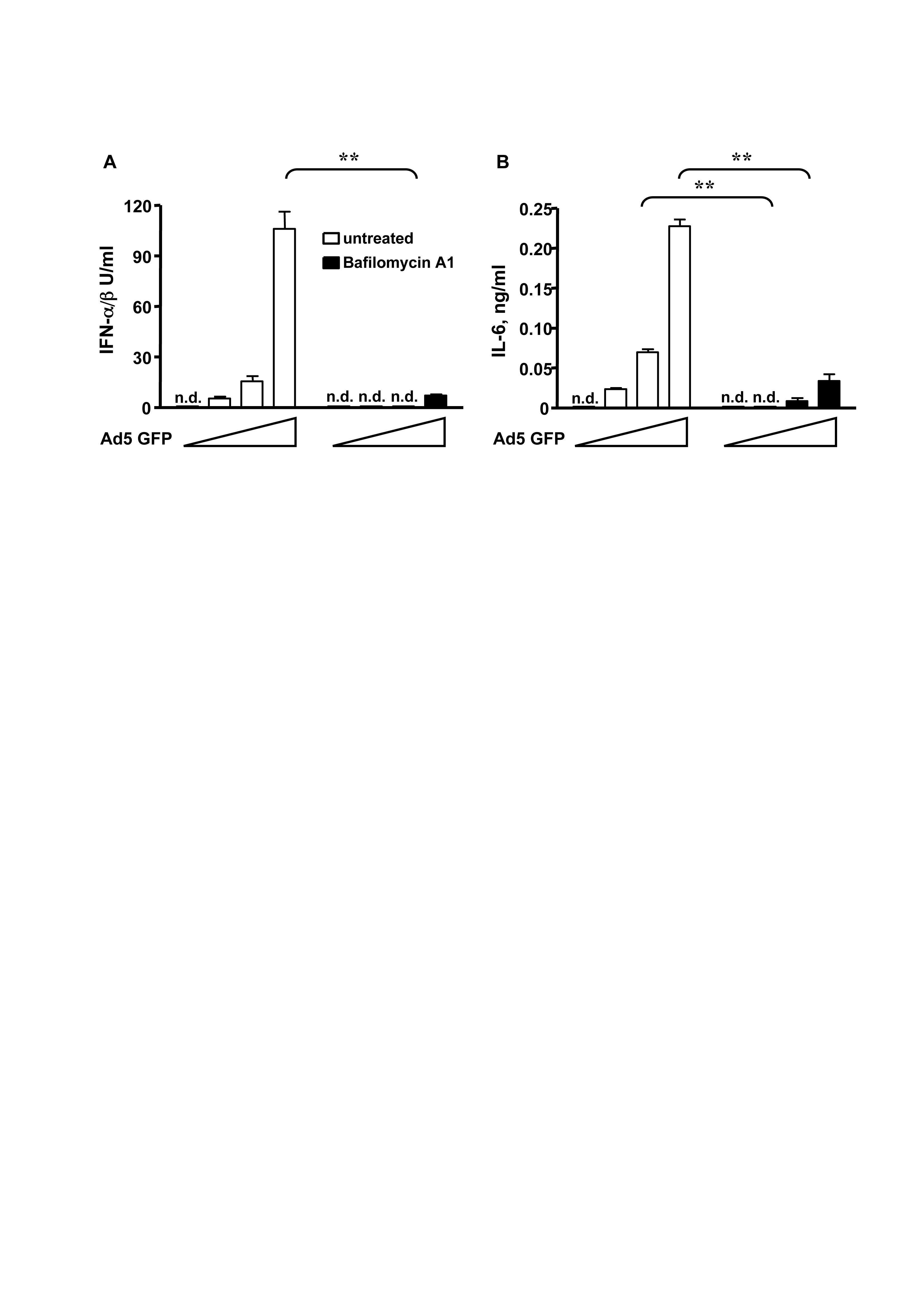

Supplement: Figure S13 — Bafilomycin A1 inhibits Ad3 triggered IFN-αβ and IL-6 production in vitro. BMDC cultures derived from C57BL/6 mice were pretreated with the 100 nM bafilomycin A1 (black bars) or with diluent (empty bars) for 15 min and then infected with 1800, 5400, and 16 200 viral particles of Ad3/cell or were mock infected. IFN-αβ (A) and IL-6 (B) levels were measured from the cell-free supernatants taken 6 h after infection. Representative experiments of three are shown. (127 KB TIF) [file ppat.1000208.s013.tif]

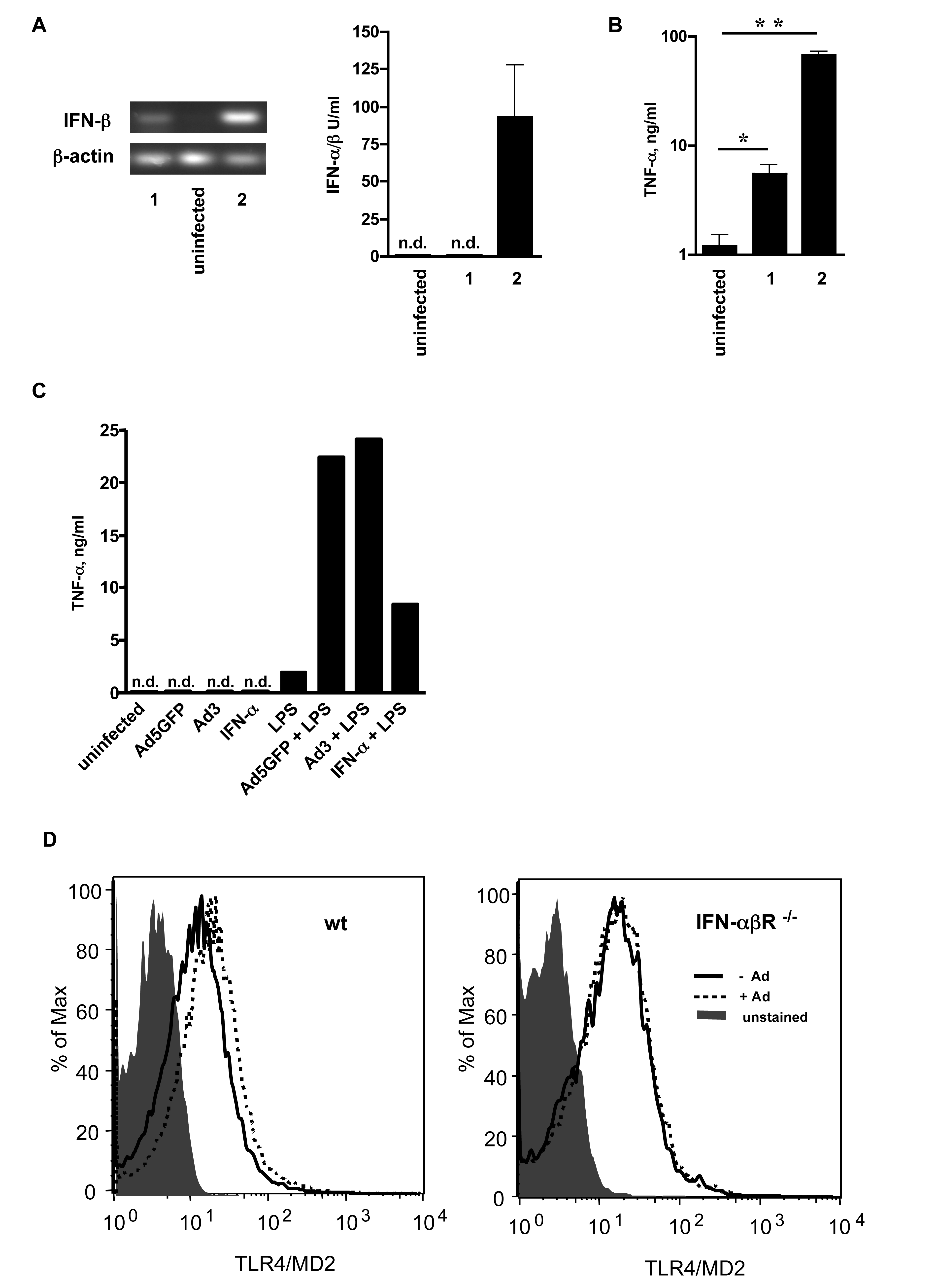

Supplement: Figure S14 — Adenovirus triggered LPS hypersensitivity. Low doses of Ads can induce LPS hypersensitivity. B6 mice (3/group) were infected with 5×10^8^ (1) or 5×10^9^ (2) particles of Ad5 GFP or left uninfected. Expression of IFN-β in spleen and plasma IFN-αβ was measured using RT-PCR and bioassay, respectively (A). In a separate experiment mice were infected as described above and 16 h later challenged with 1 µg of LPS i. p. TNF-α levels were measured in plasma 2 h later. (B). Ad infection and IFN-α treatment augments LPS induced TNF-α production of human DCs. Human monocyte derived DCs were mock-treated, or pre-infected with Ad5 GFP or Ad3 (16 200 particles/cell) or pre-treated with 50 U/ml hIFN-α. 16 h later the cells were stimulated with 1 µg/ml LPS overnight. TNF-α was measured in cell-free supernatants (C). Ad infection increases minimally the expression of TLR4/MD2 on splenic macrophages. Wt and IFN-αβR −/− mice were infected with 1×10^10^ Ad5 GFP particles i. p. or left uninfected. Expression of TLR4/MD2 was measured on the surface of F4/80+ splenic macrophages by FACS 16 h after infection (D). (335 KB TIF) [file ppat.1000208.s014.tif]
